# Choline in immunity: a key regulator of immune cell activation and function

**DOI:** 10.3389/fimmu.2025.1617077

**Published:** 2025-08-01

**Authors:** Catarina Maia, Chin Wai Fung, Elsa Sanchez-Lopez

**Affiliations:** Department of Orthopedic Surgery, School of Medicine, University of California, San Diego, San Diego, CA, United States

**Keywords:** choline, acetylcholine (ACh), choline kinase (ChoK), phosphocholine, phosphatidylcholine (PC), inflammation, immune cells, immunity

## Abstract

Nutrient availability is a strong determinant of cell function. Immune cells, which must rapidly activate transcriptional, proteomic, and metabolic programs to fulfill their functional roles, depend on nutrient supply to generate the building blocks needed for the production of immune effectors. While glucose, glutamine, and amino acids are well-recognized as critical energy sources and carbon donors during immune activation, the contribution of choline, a vitamin-like metabolite, has been overlooked. Once taken up by cells, choline plays a vital role in several biological processes. It is a precursor for phosphatidylcholine, the primary phospholipid in cellular membranes, and is also essential for synthesizing the neurotransmitter acetylcholine. Additionally, when directed toward mitochondria and betaine synthesis, choline serves as a methyl donor for histone and protein methylation, key processes that regulate gene expression and cellular activity. In this review, we examine the latest research on how immune cells utilize and metabolize choline, as well as its broader implications for immune-related disorders and overall human health. We also discuss recent and ongoing clinical studies investigating the effects of choline supplementation and the potential use of choline-derived metabolites as biomarkers for therapy response.

## 1 Introduction

Nutrient availability is essential for maintaining cellular homeostasis, supplying the building blocks necessary for cellular processes and functions. While cells can generate some nutrients through metabolic and catabolic pathways, these endogenous sources are insufficient to meet cellular demands, making dietary intake the primary source (1). In recent decades, the field of immunometabolism has significantly advanced our understanding of nutrient utilization by immune cells. Notably, studies have demonstrated the critical roles of glucose, amino acids, fatty acids, and vitamins in immune cell maturation, differentiation, and function (1, 2). However, while choline has been extensively studied in cancer due to its essential role in cell proliferation and tumor growth, its functions in immunometabolism are only beginning to be defined. Recent evidence has identified choline as a key metabolite contributing to immune cell function in various pathological settings (3, 4). Here, we will discuss the major findings on how choline availability and mobilization towards phosphatidylcholine (PC), acetylcholine (ACh), or betaine synthesis affect immune cell function, emphasizing its impact on human health and disease.

### 1.1 Choline uptake and utilization

**Figure 1:**
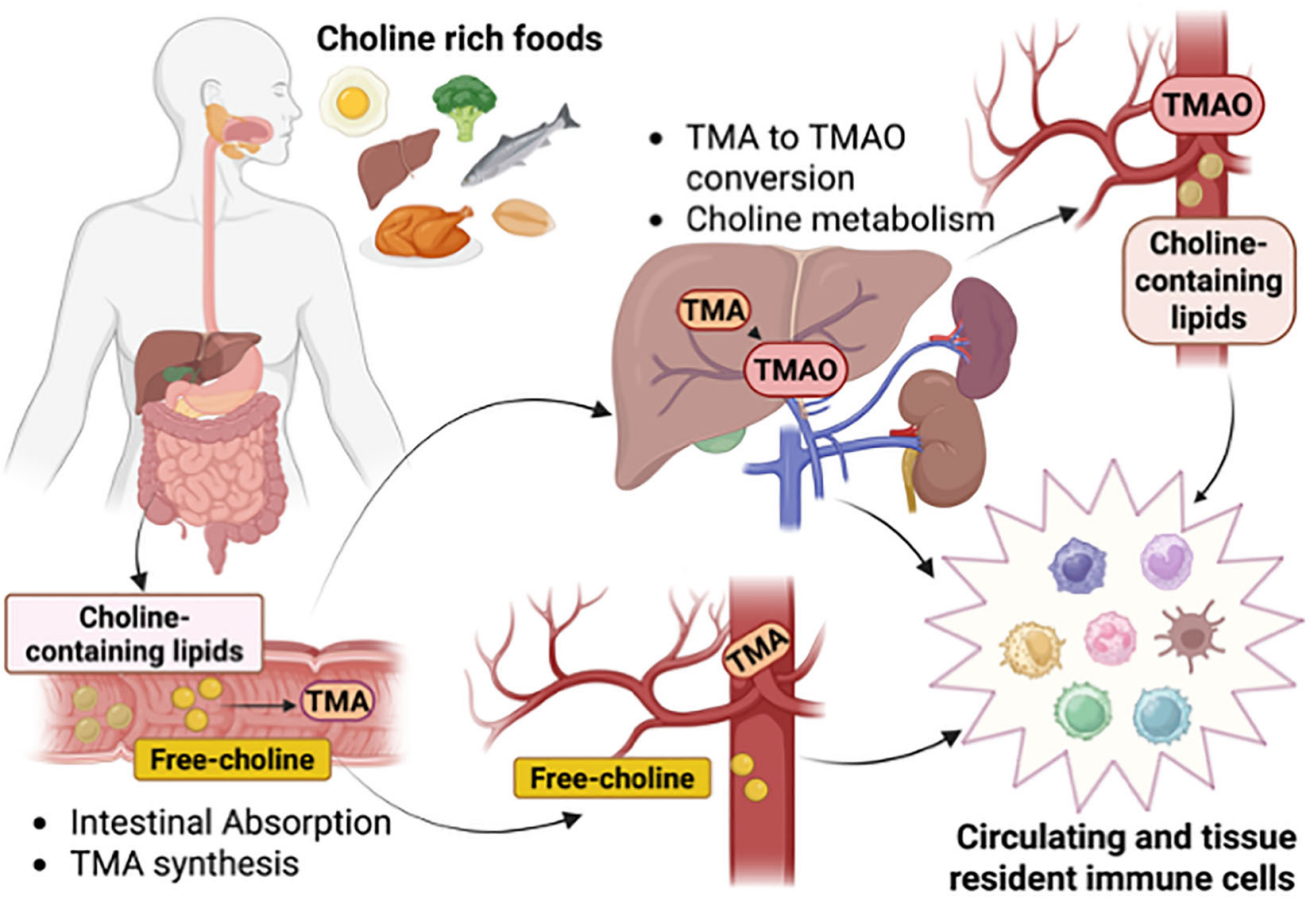
Choline intake, distribution, and metabolism. Intake of choline-rich foods provides choline bound to lipids or as a free soluble metabolite for reabsorption by intestinal cells or poured into the circulation. Intestinal bacteria uptake choline to generate the metabolite trimethylamine (TMA) that through the portal vein reaches the liver to be converted into trimethylamine N-oxide (TMAO). Circulating and tissue-resident immune cells are exposed to choline-containing lipids, free-choline, TMA, and TMAO, and respond to changes in physiological levels. Created in BioRender. Sanchez Lopez, E. (2025) https://BioRender.com/cs12pz8.

**Figure 2:**
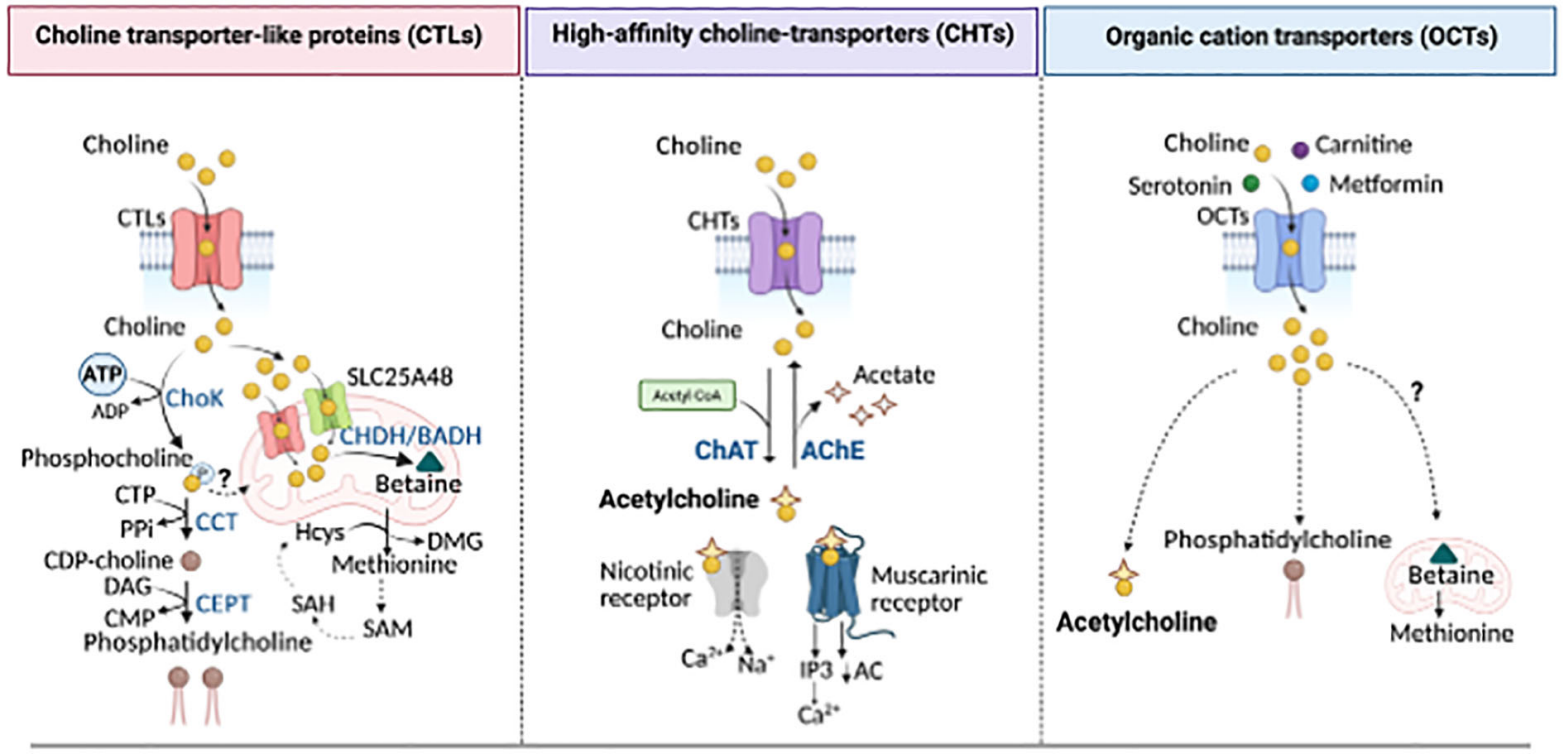
Choline transporters and utilization. Choline is transported into the cells via high- (CHTS), intermediate- (CTLs), and low-affinity (OCTs) linked to distinct metabolic fates. Left panel: CTLs mediate the uptake of choline for PC production through the Kennedy pathway. Choline is phosphorylated by Choline kinase (ChoK) to form phosphocholine, then converted into CDP-choline by the CTP:phosphocholine cytidylyltransferase (CCT). CDP-choline is converted into PC, the main phospholipid in cellular membranes, by the choline/ethanolamine phosphotransferase (CEPT). CTLs also localize to the mitochondrial membrane where, along with SLC25A48, they mediate choline import into the mitochondria for the generation of betaine, a precursor of methionine and dimethylglycine (DMG), s-adenosylmethionine (SAM), s-adenosylhomocysteine (SAH), and homocysteine (Hcys), all key methylation intermediates for DNA and protein methylation. Middle panel: High-affinity CHTS supports choline uptake for acetylcholine (ACh) synthesis through the choline acetyltransferase (CHAT), a reaction that can be reversed by acetylcholine esterase (AChE), releasing choline and acetate. Acetylcholine acts on both nicotinic and muscarinic receptors to mediate cholinergic signaling. Right panel: Low-affinity organic cation transporters contribute to choline uptake for both ACh and PC synthesis. However, their role in mitochondrial choline utilization and methionine synthesis remains unknown. PPi, inorganic pyrophosphate; DAG, Diacylglycerol; AC, adenylyl cyclase; IP3, inositol 1,4,5-triphosphate. Created in BioRender. Sanchez Lopez, E. (2025). https://BioRender.com/ahp7xe9.

Choline is a vitamin-like essential quaternary ammonium salt present in the diet as free choline and phospholipid-bound forms. Dietary choline is partially absorbed by intestinal cells or metabolized by gut bacteria (5, 6) (**Figure 1**). While choline does not compete with other nutrients for enterocyte transport, gut microbes can limit its bioavailability (7). In the large intestine, gut microbiota, including Firmicutes and Proteobacteria, convert choline into trimethylamine (TMA) (8), which is subsequently absorbed and oxidized in the liver by flavin monooxygenase 3 (FMO3) into trimethylamine-N-oxidase (TMAO) (9). In *Escherichia coli*, *Pseudomonas aeruginosa*, and *Acinetobacter baumannii*, choline uptake is mainly mediated by the BetT transporter, whose expression and transport activity are upregulated by hyperosmotic stress (10). Bacteria use choline primarily as a precursor to glycine betaine, a potent osmoprotectant (11), and as a carbon and nitrogen source (12). In contrast, in eukaryotic cells, choline transport is more complex and relies on various protein transport systems, including choline transporter-like proteins (CTLs), high-affinity choline-specific transporters (CHTs), and organic cation transporters (OCTs) (13) (**Figure 2**). The preference for a specific transport system is cell-dependent and aligns with the anticipated metabolic fate of choline. Thus, CTLs facilitate choline transport across the plasma and mitochondrial membranes, aiding in the production of phosphatidylcholine (PC) and betaine, respectively (14). CHTs mainly mediate sodium-dependent choline uptake in nervous tissues and aid the synthesis of the neurotransmitter acetylcholine (ACh) (15), while OCT2 plays a role in ACh recycling in cholinergic neurons in the presynaptic terminals (16). Recent evidence has identified mitochondrial choline import via the orphan solute carrier SLC25A48, leading to the production of betaine (17–19), a choline-derived methyl donor, and the synthesis of purine nucleotides (18–20). Loss or a single nucleotide polymorphism on the SLC25A48 gene inhibits mitochondrial choline import, increases reactive oxygen species, disrupts lipid balance, and impairs cell proliferation (18–20).

Once intracellular, choline is directed into various metabolic pathways, including 1) its oxidation to form mitochondrial betaine, 2) its acetylation to produce ACh, and 3) its initial phosphorylation followed by mobilization in the Kennedy pathway to generate cholie-containing phospholipids (**Figure 2**). Choline undergoes irreversible oxidation to betaine through a two-step process mediated by choline dehydrogenase or choline oxidase. Betaine serves as an osmolyte and methyl group donor, playing a role in the re-methylation of homocysteine into methionine, a precursor of S-adenosylmethionine (SAM), essential for numerous methylation reactions, including DNA epigenetics (21–24). In the human body, choline is primarily utilized for synthesizing essential lipids such as sphingomyelin and phosphatidylcholine (PC), which are major components of cellular membranes (25). In particular, PC accounts for approximately 95% of the total cellular choline pool in most cells. It is synthesized from choline via the Kennedy Pathway, involving cytidine diphosphate-choline and a lipid anchor such as diacylglycerol (26). *De novo* PC synthesis begins with the phosphorylation of choline by the enzyme choline kinase (ChoK) into phosphocholine (27) (**Figure 2**). Phosphocholine is then converted into cytidine diphosphate-choline by phosphocholine cytidylyltransferase (CTP), which, using either diacylglycerol or alkyl-acylglycerol as a lipid anchor, is ultimately converted into PC (26). Additionally, choline contributes to the production of lipid mediators such as lysophosphatidylcholine (lysoPC), sphingomyelin, and platelet-activating factor (28).

Beyond lipid metabolism, choline plays a critical role in non-metabolic functions. Choline is also the precursor of the neurotransmitter ACh, whose synthesis is catalyzed by choline acetyltransferase (ChAT), transferring an acetyl group from acetyl-coenzyme A to choline, resulting in ACh and coenzyme A production (**Figure 2**). After being released and bound to its receptors, ACh is rapidly hydrolyzed into acetate and choline by acetylcholinesterase. Then, free choline is transported back by CHTs for further ACh synthesis (29). Cholinergic signaling in immune cells appears to regulate cytokine synthesis, influencing both the initiation and termination of inflammation (e.g., IL-2 in T cells, TNFα in macrophages, and IL-8 in dendritic cells). Modulation of immune cell cholinergic activity, by regulating ChAT activity, ACh breakdown or choline reuptake, regulates physiological responses such as blood pressure control or anti-viral immune reaction (17, 30–34).

### 1.2 Choline demands throughout the lifespan and choline supplementation

Choline is an essential nutrient throughout life, with particularly high demands during periods of rapid cell proliferation, such as pregnancy. Choline is actively transported across the placenta, leading to fetal plasma choline levels six to seven times higher than maternal blood levels, and amniotic fluid concentrations nearly ten times higher (35). This underscores the critical role of choline in fetal development, particularly in neural tube formation and hippocampal development (36, 37). To prevent deficiency-related complications, health organizations, including the National Institutes of Health (NIH) and the European Food Safety Authority (EFSA), recommend a daily choline intake of 550 mg/day for men and 425 mg/day for women, increasing to 450 mg/day during pregnancy and 550 mg/day during lactation (22). Deficiencies during pregnancy can impair long-term potentiation and memory, while prenatal and early-life choline supplementation has been proposed as an intervention to enhance cognitive outcomes, such as improving cognitive function and mitigating age-related memory decline, enhancing long-term memory, and sustained attention (38–46). Choline supplementation (5 g choline chloride/kg) in rats during pregnancy also protects against gestational inflammation mediated by LPS challenge, reducing the frequency of loss of fetuses, normalizing placenta weights, and attenuating LPS-induced NF-κB activation and TNF-α, IL-1β, IL-6, and IL-17A levels in the placenta (47). Maternal dietary choline can be delivered to the offspring through lactation. Pups from phosphatidylcholine (PC)-fed (egg lecithin) dams have increased concentrations of PC in the plasma and spleen and a lower frequency of antigen-presenting cells (48). However, splenocytes from pups from PC-fed dams produced more IL-2, IL-6, and IFN-γ after stimulation with concanavalin A and LPS (48). Additionally, postnatal choline supplementation (100 mg/kg/day) for 20 consecutive days has been shown to mitigate the long-term effects of prenatal ethanol exposure on hippocampal inflammation and peripheral immune responses in rats (49, 50).

In females, as estrogen levels decline, the choline metabolism and utilization undergo significant changes. A study examining choline intake in 664 subjects enrolled in the cross-sectional study Nonalcoholic Steatohepatitis (NASH) Clinical Research Network (NCT00063622 and NCT00063635) showed that postmenopausal women with self-reported choline intake less than 50% of the adequate intake, had faster progression of NASH as shown by increased liver fibrosis, while no associations were found in children, men, and premenopausal women (51). Dietary choline deprivation led to fatty liver and muscle damage in 77% of men and 80% of postmenopausal women, whereas only 44% of the premenopausal women exhibited signs of organ dysfunction (52). These findings highlight that choline deficiency may become more severe in certain populations, such as postmenopausal women. This is partly due to the loss of ability to maintain the expression of genes with estrogen-response elements involved in choline metabolism. One such gene is phosphatidylethanolamine N-methyltransferase (*PEMT*) (53), which encodes the enzyme that catalyzes the conversion of phosphatidylethanolamine to PC, through a three-step methylation process using S-adenosylmethionine (SAM) as methyl donor.

In the same line with prenatal choline intake and its effect on cognition and memory, in adult individuals, sustained choline intake between 187.6-399.50 mg/day, is linked to reduced risk of cognitive decline, improved learning ability, verbal fluency, working memory, mental processing speed, and attention span, compared to individuals consuming less than 187.6 mg/kg of total choline (54). Other epidemiological studies have revealed that plasma choline levels are inversely correlated with anxiety (55) and risk of depressive symptoms (56), supporting a potential positive role of choline in mental health as well. While insufficient choline intake is linked to liver dysfunction, neurodegeneration, and muscle damage, excessive intake primarily results in mild cholinergic side effects such as sweating, diarrhea, hypotension, and fishy body odor, with a tolerable upper intake level of 3.5 g/day in adult individuals (57–59). Despite recommendations, a significant portion of the population consumes insufficient choline, and both patients with cirrhosis and those critically ill on parenteral nutrition often experience severe choline deficiencies negatively affect their outcomes (60–62). However, even while consumption levels are optimal, choline depletion may occur locally in specific disease contexts, such as within the tumor microenvironment or at sites of infection by choline-consuming pathogens (63) or systemically, such as in critically ill patients on parenteral nutrition (62). It remains uncertain whether dysregulated choline distribution affects disease progression, and significant gaps exist in understanding how cells adapt to impaired choline availability or metabolism locally.

## 2 Choline and choline-containing metabolites contribute to immune cell activation and function

Metabolic reprogramming and lipidomic remodeling are hallmarks of immune cell activation and are closely intertwined with functional responses (64–66). However, how choline utilization and phospholipid homeostasis are regulated during these metabolic shifts remains incompletely understood. Increasing evidence suggests that abnormal choline uptake and metabolism represent a metabolic hallmark associated with immune cell activation and inflammation (67–69). Disruptions in choline metabolism, whether due to impaired uptake or blockade of *de novo* PC synthesis by pharmacological inhibition of ChoK or CTP:phosphocholine cytidylyltransferase alpha (CCTα), alter the cellular phospholipid pool composition, the integrity of the mitochondrial membrane, and the overall cellular activity, leading to immune dysfunction and dysregulated inflammation across species (67, 69–72). Choline has been shown to regulate cytokine levels following lipopolysaccharide (LPS) treatment (67, 69, 70) and modulate inflammatory markers and homocysteine concentration (21). Dietary supplementation with different forms of choline has varying effects on immune system maturation, with phosphatidylcholine (PC)-rich diets demonstrating stronger immunomodulatory effects than free choline (73). For instance, supplementation of choline as PC is associated with increased T-cell proliferation, higher IL-2, IL-6, and TNF-α production (73), and supports both maternal immune function and the development of the offspring’s immune system (74, 75). However, the effects of other choline-containing lipids, such as sphingomyelin, on immune system development remain largely unexplored. Given the role of choline in immune regulation, in this section of the review, we will discuss its role across various immune cell types.

### 2.1 Myeloid cells

Myeloid cells are frontline immune defenders that restore tissue homeostasis after infection or tissue damage. Dysfunction of these cells contributes to chronic inflammatory and autoimmune diseases such as gout, rheumatoid arthritis (RA), osteoarthritis (OA), cryopyrin-associated periodic syndromes (CAPS), inflammatory bowel disease, and neurodegeneration. Their functional plasticity, shifting between inflammatory, repair, or anti-inflammatory states, depends on metabolic adaptation and lipid remodeling, including choline uptake and phospholipid reorganization (66, 76). Therefore, disrupting choline metabolism can significantly alter myeloid cell function and disease outcomes.

#### 2.1.1 Macrophages and dendritic cells

**Figure 3:**
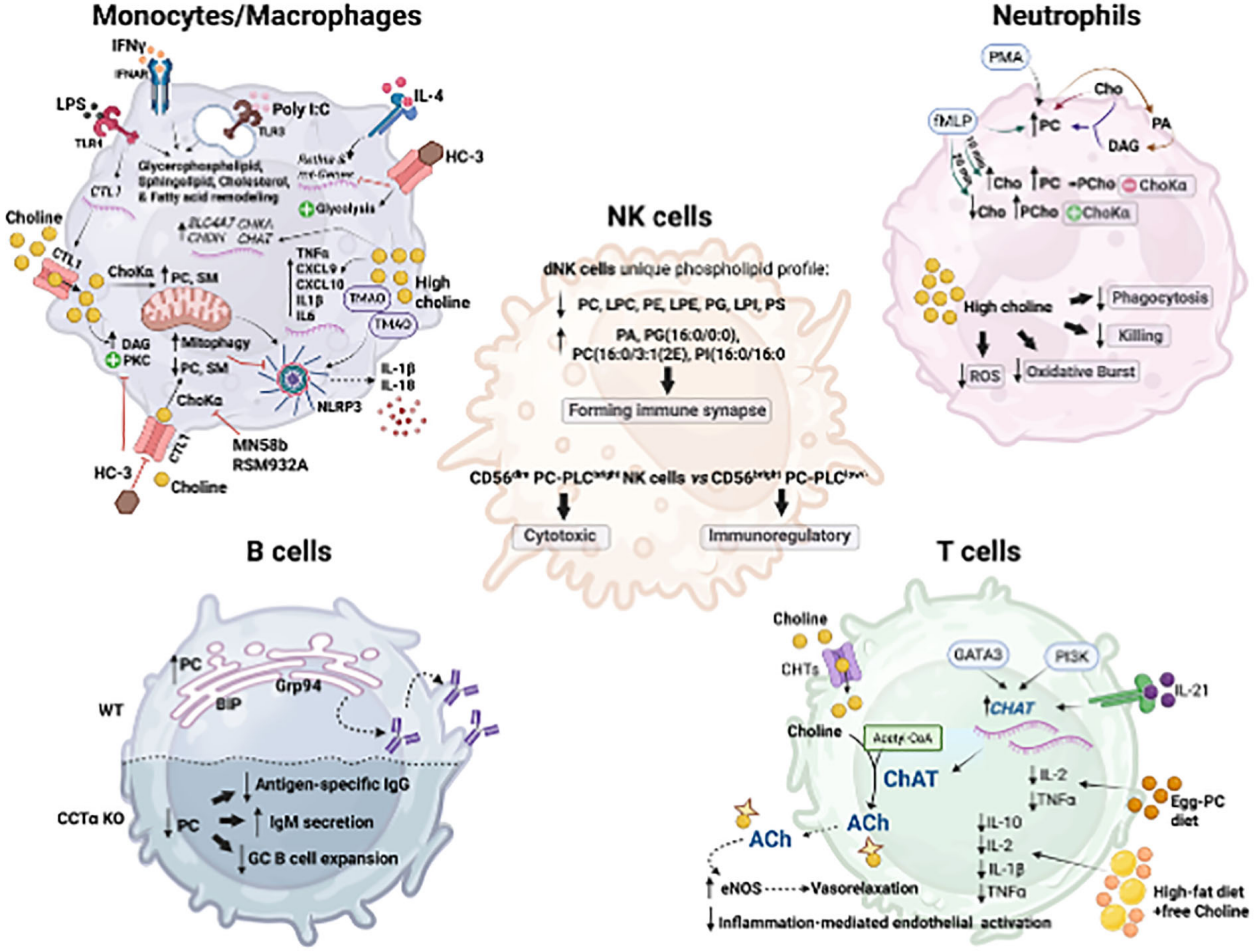
Central mechanisms of choline utilization and metabolism in the different immune cell types. Macrophage/Monocytes: Activation of macrophages (e.g., LPS, IFNy, Poly(I:C), IL-4) increases choline uptake via choline transporter-like protein 1 (CTL1) and PC synthesis. Impaired choline uptake or PCho production disrupts mitochondrial PC and SM, increasing mitophagy, restraining NLRP3 inflammasome activation and IL-1b and IL-18 production; and favors DAG accumulation and PKC activation; and suppresses IL-4-induced mitochondrial genes and Retina expression. Choline supplementation increased SLC4A7, CHDH, CHKA, and CHAT. Neutrophils: PMA and fMLP increase PC from the condensation reaction of choline and DAG formed from PC- derived phosphatidic acid. fMLP also increases PC dependent and independent of Choka activity. High choline diminishes neutrophils' phagocytic and killing capacities, reduces oxidative burst capacity, and decreases ROS production. NK cells: Human decidual NK (dNK) cells display a unique high saturated phospholipid profile compared to blood NK cells (decreased PC, LPC, PE, LPE, PG, LPI, and PS; increased PA, PG (16:0/0:0), PC(16:0/3:1 (2E)) and PI(16:0/16:0)), which difficult forming immune synapses. CD56dim PC- phospholipase C (PLC) bright NK cells are associated with cytotoxic function, whereas CD56brightPC-PLClow- cells exhibit immunoregulatory properties. B cells: Increased synthesis of PC supports rough endoplasmic reticulum (ER) expansion and upregulation of ER chaperones such as BIP and GRP94, immunoglobulin synthesis, and assembly. CCTa-deficient B cells show impaired class switching, reducing antigen-specific IgG1 production while increasing IgM secretion upon antigen challenge. T cells: Viral infection increases ChAT expression in CD4+ and CD8+ T cells in an IL21-dependent manner and via PI3K signaling cascade activation and the Th2-associated master regulator GATA3. T-cell-derived ACh boosts endothelial nitric oxide synthase (eNOS) activity and vasorelaxation and reduces inflammation. Created in BioRender. Sanchez Lopez, E. (2025). https://BioRender.com/6mvmfzs.

Macrophage functions, such as cytokine and chemokine secretion, phagocytosis, and organelle biogenesis, are intricately linked to membrane phospholipid composition, curvature, and charge (77, 78). In fact, distinct stimuli (e.g., Poly[I: C], LPS, IFN-γ, IFNβ, or IL-4) remodel the macrophage lipid composition in a signal-specific manner, altering glycerophospholipids, sphingolipids, cholesterol, and fatty acid composition (70, 79) (**Figure 3**). Choline metabolism plays a central role in macrophage activation towards both pro-inflammatory and anti-inflammatory functions (67, 69). In LPS-induced macrophage polarization, which initiates the inflammatory program, there is an augmented rate of choline uptake and PC synthesis, facilitated by the upregulation of the choline transporter CTL1 (69, 70). Pharmacologically- or antibody-mediated inhibition of choline uptake favors diacylglycerol (DAG) accumulation and protein kinase C activation, resulting in altered cytokine secretion in response to LPS (70) (**Figure 3**). Similarly, when phosphatidylcholine (PC) synthesis is compromised by myeloid-specific deletion of CTP:phosphocholine cytidylyltransferase alpha (CCTα), the rate-limiting enzyme in PC synthesis, macrophages fail to secrete the pro-inflammatory cytokines TNF-α and IL-6 in response to LPS, most likely due to secretory defects of the ER and Golgi (78). Through a distinct mechanism, choline uptake and PC synthesis regulate IL-1β production mediated by NLRP3 inflammasome activation (69) (**Figure 3**). Macrophages exposed to choline deficiency or ChoKα inhibitors (MN58b or RMS932A) exhibit poor PC and sphingomyelin mitochondrial membrane composition, leading to defective mitochondrial ATP synthesis that boosts AMPK activation and mitophagy (**Figure 3**). This eventually prevents mitochondrial damage and the cytosolic release of oxidized mtDNA, a direct activator of NLRP3, limiting the processing of mature IL-1β^69^. Similarly, both *in vivo* feeding with a choline-rich diet and *in vitro* trimethylamine-N-oxidase (TMAO) stimulation increased TMAO-dependent NLRP3 inflammasome activation and expression of IL-1β, IL6, TNF-α, CXCL9, and CXCL10, supporting that NLRP3 is a key proteolytic activator in the macrophage response to a high choline and TMAO production (80) (**Figure 3**). Indeed, in a model of graft-versus-host disease (GVHD), T-cell-depleted bone marrow transplants fed with TMAO-rich diet exhibited a significant increase in the frequency of F4/80^+^CD11b^+^CD16/32^+^ inflammatory macrophages relative to the F4/80^+^CD11b^+^ whole population, worsening disease severity, and increasing mortality (80).

Beyond the inflammatory response, choline metabolism is also implicated in IL-4-induced macrophage polarization, critical for immunity against intestinal helminth infection, resolution of inflammation, and tissue repair. IL-4 causes a rapid increase in choline import, phosphocholine production, and PC biosynthesis (67). The inhibition of choline transport and metabolism, using hemicholinium (HC-3), and RSM932A, which target the choline transporter and ChoKα, respectively, dramatically impacted macrophage responses to IL-4. Specifically, inhibition of choline metabolism suppresses mitochondrial gene expression, shifts metabolism toward glycolysis, and inhibits IL-4-induced expression of *Retnla* (67), a resisting-like molecule involved in type 2 immunity and tissue repair. In a model of intestinal infection with the parasite Heligmosomoides polygyrus, ChoKα inhibition diminished peritoneal macrophage and B-1 lymphocyte frequency, causing compromised immunity against the parasite (67). In the human monocytic cell line U937, IL-4 receptor engagement leads to ChoK-independent increases of phosphocholine, resulting from the degradation of membrane PC into DAG, indicating enhanced PC-specific phospholipase C activity (81). Choline availability may also regulate and support the expression of the machinery necessary for its processing and signaling, as dietary choline supplementation increases the expression of choline receptor *SLC4A7*, choline dehydrogenase (*CHDH*), choline kinase alpha (*CHKA*), choline acetyltransferase (*CHAT*), and genes related to acetylcholine (ACh)-dependent signaling such as the muscarinic (*CHRM1* and *CHRM5*) and nicotinic (*CHRNA7*) ACh receptors (82) (**Figure 3**). Indeed, small peritoneal macrophages maintain peritoneal ACh levels through choline acetyltransferase expression driven by MyD88 pathway activation. This increased macrophage-mediated acetylcholine (ACh) release facilitates the clearance of apoptotic neutrophils and enables the resolution of acute peritonitis (83). In addition, intestinal macrophages respond to ACh released by nerve fibers in the intestinal myenteric plexus, ameliorating inflammation *in vivo*, in a mechanism that involves the activation of Jak2/STAT3 and the transactivation of STAT3-responsive DNA elements (84). The stimulation of the vagus nerve in rats at 10 *Hz*, suppresses endotoxin-induced serum TNF-α levels *in vivo*. Similarly, *in vitro* exposure of macrophages to 10 μM of ACh prior to endotoxin challenge reduces TNF-α production by increasing adenylyl cyclase 6 activity, leading to cAMP formation, CREB phosphorylation, and the expression of c-Fos, a known inhibitor of TNF transcription (85). This inhibitory effect on monocytes’ TNF and IL-1β production was confirmed in human whole blood and human monocyte/macrophages using a selective inhibitor of α7 nicotinic acetylcholine receptor (α7nAChR) (86). Together, all these findings emphasize the essential role of choline availability in macrophage preparedness for an efficient immune response to diverse triggers and reveal that the impact of impaired choline metabolism and utilization on inflammatory molecular pathways is cell- and stimulus-specific.

It is worth noting that the different choline dietary forms differ in their effect. The offspring from dams fed with mixed choline sources with lower free choline (12.5-25%) but high glycerophosphocholine (25-75%), exhibited a decrease in macrophage and dendritic cell frequencies in the spleen and produced less IL-1β, IL-6 and IFN-γ in response to mitogenic immune challenge with either concanavalin A or LPS, compared to those from dams with higher free choline (100%) intake (87). In the context of obesity-related inflammation, adipose tissue macrophages isolated from obese mice and humans often exhibit increased *de novo* PC biosynthesis (71). Interestingly, macrophage-specific deletion of CCTα alleviated obesity-induced white adipose tissue (WAT) inflammation and insulin resistance. However, despite reduced CCTα activity, PC levels remain unchanged due to a compensatory reduction in PC degradation, resulting in slower PC turnover that allows for greater remodeling of PC species enriched in polyunsaturated fatty acid (PUFA), which likely protects against endoplasmic reticulum stress and inflammation (71). These establish a causal relationship between obesity-associated increases in the *de novo* PC synthesis, accelerated PC turnover, and proinflammatory adipose tissue macrophage activation (71).

In dendritic cells, ACh modulates immune function by upregulating HLA-DR and CD86 expression and stimulating TNF-α and IL-8 production (34). However, when combined with LPS stimulation, ACh partially suppresses HLA-DR and TNF-α/IL-12 production, suggesting that its effect depends on dendritic cell maturation status (34). Despite these findings, the influence of choline metabolism on dendritic cell adaptation, particularly in high-choline-demand environments such as tumors, remains unexplored. The gut microbiota also contributes to choline-mediated immune modulation. *Enterobacter ludwiggi*, an abundant commensal bacterial species in mice, enhances dendritic cell immune tolerance and promotes Treg differentiation via choline metabolism (88). Choline produced by *E. ludwiggi* protects against DSS-induced colitis by enhancing the choline/α7nAChR-mediated dendritic cell immune tolerance, leading to increased Foxp3^+^ Treg differentiation (88). CD103^+^ dendritic cells from *Enterobacter ludwiggi*-treated mice exhibited higher expression of tolerogenic markers *Tgfb1, Tgfb2, Aldh1a2*, and *Pdl1*, and co-culture with naïve CD4^+^ T cells enhances Treg conversion (88). Moreover, mice receiving dendritic cells exposed to *Enterobacter ludwiggi* exhibited reduced colitis severity and expanded Treg populations in the mesenteric lymph nodes and spleen, highlighting choline’s role in shaping gut-immune interactions.

Together, these findings highlight the complex role of choline metabolism in shaping antigen-presenting cell function and inflammatory responses across diverse physiological and pathological contexts. A deeper understanding of how choline-derived metabolites influence innate immune signaling may uncover new therapeutic targets and biomarkers for inflammatory disease management and precision immunomodulation.

#### 2.1.2 Neutrophils and eosinophils

Granulocytes play a pivotal role in the initial immune response to pathogen infection or tissue damage, contributing to both inflammation and tissue homeostasis. Among them, neutrophils are the most abundant and are essential for detecting pathogens and initiating immune cascades through processes such as swarming, cytokine production, degranulation, phagocytosis, and the formation of neutrophil extracellular traps (NET) (89, 90). Two decades ago, Tronchère et al. described that neutrophil activation by Phorbol 12-myristate 13-acetate (PMA) and formyl-methionyl-leucyl-phenylalanine (fMLP) results in increased choline incorporation into phosphatidylcholine (PC), dependent solely on diacylglycerol formed from PC-derived phosphatidic acid (91) (**Figure 3**). This provides evidence for an activated PC cycle in human neutrophils linking phospholipase D and cytidyltransferase activation. However, these findings were later challenged by Pédruzzi et al. (92), who showed that while fMLP and PMA stimulation altered choline and PC levels in neutrophils, phosphorylcholine content remained unchanged for at least 10 minutes, indicating that phospholipase C-mediated PC breakdown was not the primary mechanism. Notably, prolonged fMLP exposure (20 minutes) increased phosphocholine while decreasing choline levels, implicating ChoKα activity rather than phospholipase C-mediated PC degradation (92) (**Figure 3**). Interestingly, PMA stimulation showed a distinct response, showing a decline in phosphocholine between 10 and 15 minutes, indicating a stimulus-dependent, distinct regulatory mechanism of phosphocholine metabolism (92). Despite early evidence linking choline and PC metabolism to neutrophil activation, the broader impact of choline and its metabolites on neutrophil function remains poorly understood. Studies on bovine neutrophils suggest that increasing choline concentrations linearly diminish their phagocytic and killing capacities (82). In neutrophils from early lactation cows, dietary choline supplementation increased the expression of SLC5A7, CHDH, CHKA, CHAT, CHRM5, and CHRNA7. Choline significantly reduced neutrophil oxidative burst capacity in a dose-dependent manner, and the higher choline concentration (13.2 μM) led to a quadratic decrease in *E. coli* phagocytosis and a linear reduction in reactive oxygen species (ROS) production per neutrophil (82). Inflammatory gene expression, including *TLR4, NFKB1, TNFA*, neutrophil elastase *(ELANE), H2A, CASP3*, and *CASP7*, was largely unaffected by choline, though a greater reduction in *TLR4* expression was observed at higher doses. TNFα levels also tended to decrease following choline supplementation (**Figure 3**).

Eosinophils contribute to various inflammatory conditions, including asthma, rhinitis, eosinophilic gastroenteritis, and inflammatory bowel disease. While choline exhibits anti-inflammatory activity, its role in eosinophilic inflammation remains unclear. Dietary choline supplementation effectively suppressed airway inflammation in an allergen-induced mouse model of airway hyperreactivity by reducing eosinophil accumulation and eosinophilic peroxidase activity in bronchoalveolar lavage fluid, likely through nicotinic acetylcholine receptor activation via the cholinergic anti-inflammatory pathway (93). Later studies by the same group confirmed these findings, showing that co-administration of choline and α-lipoic acid further reduced eosinophilic infiltration, peroxidase activity, and oxidative stress, suggesting a role for redox status modulation (93). Similarly, asthma patients receiving conventional therapy alongside six months of oral choline supplementation exhibited significant reductions in circulating eosinophils and factors involved in bronchial hyperreactivity, such as IL-5 and cysteinyl leukotrienes (Cys-LT) (94). Choline, as a precursor of PC, a major component of lung surfactants, may help compensate for eosinophilic phospholipases-pulmonary surfactant dysfunction in asthma, thereby reducing airway inflammation and disease severity (95). However, further mechanistic studies are needed to determine the direct effects of choline availability on eosinophil activation and function and to assess the therapeutic potential of dietary choline supplements (95) in managing hyperreactive eosinophilic conditions, such as eosinophilic myositis or gastrointestinal disorders.

#### 2.1.3 Microglia

Microglia constitute specialized immune sentinels of the central nervous system that exhibit similar plasticity to macrophages, transitioning between inflammatory and anti-inflammatory phenotypes. Under stress conditions, activated microglia release inflammatory factors that drive neuroinflammation, whereas in a tissue repair state, they promote neuroprotection (96). The stimulation of the α7nAChR has been shown to exert anti-inflammatory effects by inhibiting cytokine production and release, such as TNF-α, which is neurotoxic, by microglia (97, 98). However, the role of choline uptake and the generation of choline-derived metabolites in microglia-mediated neuroinflammation remains incomplete. Besides acetylcholine (ACh) receptors, microglia also expressed choline transporter CTL1, primarily located in the plasma membrane, facilitating extracellular choline transport, and CTL2 mainly in the mitochondria, suggesting a role in betaine production (99). Microglia activation with either LPS or IL-4 enhances choline uptake and phosphatidylcholine (PC) synthesis. Notably, CTL1 inhibition or choline deprivation suppresses LPS-induced IL-1β and IL-6 but boosts the expression of arginase-1 upon IL-4 stimulation (99). This indicates that, as in macrophages, choline metabolism also modulates microglial inflammatory responses, and manipulating choline metabolism may promote a neuroprotective phenotype. Furthering this concept, research on licochalcone E (Lico E), a β-amyloid aggregation inhibitor, has revealed that CTL1-mediated choline uptake is involved in its neuroprotective effects (100). While stimulation of the microglia cell line SIM-A9 with Aβ1*–*42 significantly increased TNF-α mRNA expression, this effect was suppressed by choline deficiency and Lico E treatment. Furthermore, Lico E also restored arginase 1 expression, supporting its neuroprotective role. Additionally, IL-4-induced *Arg1* expression was further upregulated by choline deprivation and Lico E treatment, reinforcing the hypothesis that CTL1 inhibition fosters a neuroprotective anti-inflammatory phenotype promoting Aβ degradation (100).

The role of choline metabolism in inflammation extends beyond microglia. In acid sphingomyelinase knock-out mice, which mimic a neurovisceral acid sphingomyelinase deficiency (ASMD) characterized by cellular accumulation of sphingomyelin, the effects of a choline-free diet decreased the activation of liver macrophages and microglia, but it did not significantly alter sphingomyelin levels due to compensatory mechanisms involving methionine metabolism (101). While in this model, choline deprivation altered lipid composition in the liver, decreasing sphingomyelin and specific glycerophospholipids with 34:1 fatty acids, leading to reduced inflammation, its impact on brain lipid metabolism was less pronounced (101). This suggests that additional dietary modifications, such as methionine restriction, may be needed to modulate neuroinflammation more effectively. Although growing evidence highlights the impact of choline metabolism on macrophages and microglia, there is a knowledge gap regarding its effects on other tissue-resident immune sentinels, such as Kupffer and Langerhans cells. Further research is necessary to elucidate the broader implications of choline metabolism in immune regulation and inflammatory diseases.

### 2.2 Lymphocytes

The phospholipid remodeling and cholinergic anti-inflammatory system play a well-established role in immune homeostasis and the regulation of inflammatory and autoimmune diseases. Most immune cells, including CD4^+^ T cells, B cells, and NK cells, upregulate genes related to the cholinergic system synthesis in response to inflammatory cues, which is pivotal for the maintenance of immunological homeostasis (31–33, 86, 102). Below, we summarize studies that remark the importance of the cholinergic immune signaling and circulating choline-containing lipids on lymphocyte differentiation, activation, and function.

#### 2.2.1 B cells

B cell maturation and differentiation into plasma cells require robust phospholipid synthesis and remodeling to sustain multiple rounds of proliferation, clonal expansion, and antibody production (103). A hallmark of this differentiation process is the increased synthesis of phospholipids, particularly phosphatidylcholine (PC) (104), which supports the expansion of the intracellular membrane network, including the rough endoplasmic reticulum, where immunoglobulins are synthesized and assembled (105, 106) (**Figure 3**). Plasma cell differentiation is accompanied by endoplasmic reticulum enlargement, increased membrane-bound ribosomes, upregulation of endoplasmic reticulum chaperones such as BIP and GRP94, and transcription factors like XBP-1 (106). The inhibition of choline mobilization through the Kennedy pathway has been examined using B cell-specific CTP: phosphocholine cytidyltransferase (CCTα, Pcyt1a gene) deficient mice. Despite that low penetrance, partial deficiency of CCTα in B cells resulted in decreased peritoneal and splenic B cell numbers, inducing a compromised proliferation, especially in the periphery, decreasing serum IgG concentration, and increasing the incidence of IgM-secreting cells (107). CCTα-deficient B cells stimulated with LPS triggered early activation of the unfolded protein response (UPR)-mediated splicing of Xbp-1, and impaired class switching, reducing antigen-specific IgG1 production while increasing IgM secretion upon antigen challenge (104) (**Figure 3**). This highlights the requirement of PC synthesis for germinal center B cell expansion and antibody production (76, 104).

Various immune cells, such as T and B lymphocytes, and Natural Killer (NK) cells possess all the necessary components to constitute an independent cholinergic system, including ChAT and acetylcholinesterase, and both muscarinic and nicotinic ACh receptors (31, 34, 83, 108–113). In particular, the production of acetylcholine (ACh) by choline-acetyltransferase (ChAT)-expressing B cells, which seems to be essential for efficient liver regeneration in a mouse model of partial hepatectomy (112). B-cell specific ablation of ChAT increases the mortality of mice subjected to partial hepatectomy compared to their wildtype counterpart, due to dysregulation of α7nAChR expressing Kupffer cells and CD8^+^ T cells, limiting their regenerative capacity and producing harmful IFN-γ, respectively (112). Similarly, ACh-producing B cells contribute to the regulation of TNF-α production by α7nAChR-expressing interstitial lung macrophages in mice subjected to influenza infection (113). Altogether, these findings support antibody-independent immune regulatory functions of B cells and expand the immunomodulatory mechanisms associated with ACh production.

Aberrant B cell expansion and survival contribute to B cell malignancies, such as multiple myeloma, diffuse large B cell lymphoma, and chronic lymphocytic leukemia, which are frequently associated with TRAF3 gene deletions or inactivating mutations (114–116). Recent studies identified TRAF3 as a regulator of key metabolites, lipids, and enzymes involved in choline metabolism (117). In particular, TRAF3 ablation boosts phosphocholine and PC biosynthesis, promoting B cell survival (117). Pharmacological inhibition of ChoKα by MN58B and RSM932A effectively reduced the survival of TRAF3-deficient B cells both *in vivo* and *in vitro (* 117). Metabolomic, lipidomic, and transcriptomic studies indicate that TRAF3 exerts broad regulatory effects on B cell metabolism, including interconnecting choline and ethanolamine pathways. Reconstitution of TRAF3 in human multiple myeloma cell lines inhibited ChoKα expression, suppressed the Kennedy pathway, and induced apoptosis, underscoring the role of elevated choline metabolism in sustaining the phenotype of TRAF3-deficient malignant B cells.

#### 2.2.2 T cells

T follicular helper (T_FH_) cells display distinct lipid metabolic profiles, characterized by the localization of phosphatidylethanolamine predominantly on the outer plasma membrane. Similar to B cells, phosphatidylethanolamine colocalizes with the chemokine receptor CXCR5 (118). *De novo* phosphatidylethanolamine synthesis via the cytidine diphosphate-ethanolamine pathway is critical for maintaining CXCR5 surface expression by preventing its internalization and degradation. T cell-specific genetic ablation of *Pcyt2*, which encodes CTP: phosphoethanolamine cytidylyltransferase, but not of *Pcyt1a*, which mediates the cytidine diphosphate-choline pathway, impairs T_FH_ cell differentiation, leading to diminished humoral immune responses (118). Indeed, splenocyte incubation with lysoPC, a circulating bioavailable form of PC, enhances proliferation and IL-2 secretion (48), suggesting that PC positively modulates T-cell function and may counteract immune dysfunction.

T cell-derived acetylcholine (ACh) has long been studied as a crucial immune regulator. T and B cell lymphocytes express choline acetyltransferase (ChAT), high-affinity choline transporters (ChRM and ChRN), acetylcholine transferase, and can produce and release ACh (102, 119, 120). *In vitro* studies show that ACh and other ChRM and ChRN agonists enhance T cell cytotoxicity, B and T cell intracellular Ca^2+^, c-fos expression, nitric oxide, and IL-2 production, cyclic guanosine monophosphate (cGMP), and inositol-1,4,5-triphosphate (IP_3_) levels, and modulate DNA synthesis and cell proliferation (102, 119, 121–123). As mentioned above, ChAT-expressing T cells affect blood pressure (30) and regulate the release of inflammatory cytokines (33). During lymphocytic choriomeningitis virus (LCMV) infection, ChAT expression is strongly induced in both CD4^+^ and CD8^+^ T cells in an IL-21-dependent manner (31) (**Figure 3**). Using ChAT-GFP reporter mice allowed for tracking a massive expansion of CD4^+^ and CD8^+^ T cells at day 8 post-infection, followed by a rapid decline of Chat-GFP^+^ splenic virus-specific T cells after LCMV clearance (31). In this context, IL-21, a key cytokine in chronic infection, drives ChAT expression in T cells, facilitating their migration into infected tissues. In human T cells, ChAT mRNA expression is induced via the activation of the PI3K signaling cascade (124). In particular, ChAT expression is induced by the Th2-associated master regulator GATA3 and the suppression of RE-1 silencing transcription factor (REST)-mediated methylation of the ChAT promoter (124) (**Figure 3**). T cell-derived ACh potentiates endothelial nitric oxide synthase (eNOS) activity, facilitating vasorelaxation, improving endothelial barrier integrity, and reducing inflammation-associated endothelial activation (124). Furthermore, in a cohort of patients with severe circulatory failure, improved survival positively correlated with their relative frequency of circulating ChAT^+^CD4^+^ T cells (124).

Supplementation with dietary choline from egg-phosphatidylcholine (PC) mitigates T-cell dysfunction associated with diet-induced obesity (125, 126). The egg-PC diet lowered the frequency of CD3^+^ T cells with no significant differences in helper (CD3^+^CD4^+^) and cytotoxic (CD3^+^CD8^+^) T cells as well as activated T cells (CD3^+^CD25^+^), reducing as well the production of IL-2 and TNF-α 125) (**Figure 3**). In a follow-up study (125), high-fat diet supplementation with free choline exhibits a reduction in splenocyte T-cell proliferation following stimulation with anti-CD3/anti-CD28, and decreased production of IL-1β, IL-2, IL-10, and TNF-α in splenocytes and mesenteric lymph nodes (126) (**Figure 3**). Diet supplementation with mixed choline sources, with low free choline but high glycerophosphocholine, increases the frequency of cytotoxic CD8^+^ T cells expressing CD27, CD71, and CD127, as well as total B cells (CD45RA^+^) and dendritic cells (OX6^+^OX62^+^) (87). Additionally, pups of choline-supplemented dams exhibited lymphocytes that produced lower IL-6 and IFN-γ following concanavalin A stimulation, compared to those from the 100% free choline group (87).

#### 2.2.3 NK cells

Natural killer (NK) cells are innate lymphocytes that contribute to the immune response against malignancies and viral infections. Unlike the T and B cells, NK cells do not undergo receptor gene rearrangement, making them a key component of the first line of immune response.

NK cells exhibit strong metabolic flexibility upon short-term cytokine stimulation, but prolonged activation leads to increased mitochondrial metabolism and glycolysis involving mammalian target of rapamycin complex 1 (mTORC1) (127, 128). Activated NK cells rely on enhanced glycolysis and the citrate-malate shuttle, which facilitates glucose-driven mitochondrial citrate export to the cytosol. There, citrate is converted to malate before re-entering the mitochondria to fuel the electron transport chain (129). Additionally, citrate serves as a precursor for acetyl-CoA, which is essential for lipid synthesis and protein acetylation. While choline uptake and utilization in NK cells remain unexplored, choline-containing lipids play a crucial role in NK cell cytotoxicity and function.

Metabolomic and lipidomic studies have identified distinct alterations in glycerophospholipids among different NK cell subsets (130). Notably, human decidual NK (dNK) cells, which are located at the maternal-fetal interface, display a unique PL profile compared to blood NK cells (130). In dNK cells, total phosphatidylcholine (PC), lysoPC, phosphatidylethanolamine, lysophosphatidylethanolamine, phosphatidylglycerol, lysophosphatidylinositol, and phosphatidylserine are significantly downregulated (**Figure 3**). In contrast, metabolites such as phosphatidic acid, phosphatidylglycerol (16:0/0:0), PC (16:0/3:1(2E)), and phosphatidylinositol (16:0/16:0) are significantly upregulated, suggesting a high saturation of glycerophospholipids. This metabolic shift may indicate difficulty in forming immune synapses, which are critical for NK cell function (130). Intratumoral NK cells exhibit reduced sphingomyelin content, and inhibition of sphingomyelin synthase 1, the enzyme responsible for converting ceramide and PC into sphingomyelin, disrupts NK cell function (131). Since sphingomyelin is necessary for immune synapse formation, its inhibition impairs NK cell recognition and killing of tumor cells by altering membrane topology and cytotoxicity (131). A reduction in choline availability to the intratumoral NK cells may have effects similar to sphingomyelin biosynthesis inhibition, though this possibility remains unexplored. Moreover, it remains unknown whether increased PC synthesis in NK cells is mediated by choline mobilization to the Kennedy pathway or through increased PC-PLC activity. Both NK-cell-mediated cytotoxicity and lytic granule exocytosis require an increase in PC-PLC (132). Among the NK cells subset, CD56^dim^ PC-phospholipase C^bright^ cells are associated with cytotoxic function, whereas CD56^bright^ PC-phospholipase C^low/-^ cells exhibit immunoregulatory properties (133) (**Figure 3**). Indeed, PC-phospholipase C expression on the NK cell membrane correlates closely with CD16 receptor expression, suggesting a potential relationship between enzyme externalization, NK cell maturation, and CD16-mediated cytolytic process (134). The mechanistic underpinnings of choline-mediated regulation of lymphocytes, including B, T, and NK cells, remain insufficiently defined. There are significant gaps in our understanding of how choline metabolism integrates with lymphocyte function in acute and chronic inflammatory conditions.

## 3 The influence of choline on health and disease outcomes

Although the human body can synthesize choline in the liver, this endogenous production is insufficient to meet physiological demands, making dietary supplementation necessary. Choline is primarily obtained from animal-derived foods (e.g., meat, dairy, eggs), as well as plant-based sources (e.g., beans, nuts, seeds). Imbalances in choline intake, whether insufficient or excessive, can lead to various health issues, including cardiovascular disease, neurological disorders, and organ dysfunction (3, 22, 23, 135, 136). Moreover, elevated circulating or local choline metabolism has been observed in inflammatory diseases, such as arthritis, cancer, and cardiovascular diseases (68, 137–141). The knowledge gained from *in vitro*, preclinical, and animal studies has encouraged a variety of clinical trials investigating the role of dietary choline supplementation in disease progression (**Table 1**), as well as the association between circulating choline-containing metabolites and disease prognosis or clinical symptoms (**Table 2**). However, there are few studies investigating the impact of choline intake on immune responses in healthy individuals. A cross-sectional survey that enrolled healthy men (n=1514) and women (1528) who reported intakes of choline and betaine calculated from food-frequency questionnaire and food-composition tables found that participants who consumed more than 310 mg/d had lower plasma C-reactive protein (CRP), IL-6 and TNF-α than participants reporting choline intake below 250 mg/d (142). In subjects (n=33) with adequate choline intake (500 mg choline/70 kg body weight), a shift to a low-choline diet (<50 mg/d) for 42 days led to substantial transcriptomic changes in peripheral lymphocytes (152 down- and 107 up-regulated genes), providing a unique signature (including *CHEK1*, *GBE1*, and *KIF20A*), that could be used for segregating the participants according to the absence or presence of signs of organ dysfunction caused by the low choline intake, including fatty liver or elevated plasma CPK (143 *).* Importantly, these diet-induced changes in gene expression profiles were influenced by SNPs within the genes involved in choline metabolism, such as *MTHFD1, CHDH*, and *PEMT* (143). This is important, as the prevalence of SNPs in genes that increase susceptibility to choline-related organ dysfunction in the general population is not known. A parallel study revealed that all participants fed with a low choline diet had significant lymphocyte DNA damage compared to the phase in which they were fed with adequate choline amounts. The increase in lymphocyte apoptosis was more pronounced in participants fed with low diet who developed organ dysfunction (144). The impact of choline intake on transcriptional regulation has also been observed in a 12-week controlled feeding study, in 26 healthy third-trimester singleton pregnant women and 21 non-pregnant control women fed with either 480 or 930 mg total choline/day (NCT01127022) (145). In particular, the epigenetic mark histone 3 lysine 4 di-methylation (H3K4me2), which has been associated with increased transcription, was lower among pregnant women consuming higher levels of choline (145). However, further studies are needed to identify the specific genes regulated by H3K4me2 that are sensitive to choline availability.

**Table 1:** Human studies evaluating the effect of dietary choline.

Study	Participants	Intervention/Assesment	Outcome	Follow-up (months)	Clinical trial #	Ref.
Cross-sectional survey.	n=1514 men and n=1528 women; healthy adults.	Food-frequency questionnaire and food-composition tables. Self-reported choline intake.	>310 mg/d had lower plasma CRP, IL-6 and TNF-α than choline intake below 250 mg/d.	–	–	(135)
Low-choline diet.	n=33; healthy adults.	Subjects with adequate choline intake (500 mg/70 kg body weight) shift to a low choline diet (<50 mg/day) for 42 days.	Low choline diet led to transcriptional changes in peripheral lymphocytes. Gene signature associated with the presence or absence of organ dysfunction (fatty liver and elevated plasma CRP). SNPs in *MTHFD1, CHDH* and *PEMT* influence the diet-induced changes in gene expression profile.	–	–	(143)
Controlled feeding study.	n=26 healthy third-trimester singleton pregnant women; n=21 healthy non-pregnant women.	Groups: 1) 480 mg total choline/day; 2) 930 mg total choline/day; for 12 weeks.	Choline intake over the recommended adequate intake does not alter blood leukocyte count, but reduced H3K4me2.	–	NCT01127022	(145)
Dietary Supplementation. Double-blind, placebo-controlled.	n=30; asthmatic; >65 y.o.	Groups: 1) 310 mg choline bitartrate; 2) placebo; b.i.d. over 6 weeks.	No change in eosinophil count or total IgE serum levels.	–	NCT02371993	(199)
Dietary Supplementation. Double-blind , pplacebo-controlled.	n=96; diabetic; adults.	Groups: 1) choline; 2) magnesium; 3) choline + magnesium; for 2 months.	The Choline + Magnesium group exhibits decreased levels of plasma IL-6 and VCAM1.	–	IRCT20110123005670N25	(148)
Controlled feeding trial, randomized.	n=11 men; n=17 women; 65+ y.o.	Groups: 1) 3 oz, or 2) 6 oz, of lean fresh beef within the standardized Dietary Approaches to Stop Hypertension (DASH) diet, for 12 weeks. Fasted blood serum samples for metabolomic analysis.	Beef intake decreases plasma choline, dimethylglycine, PC, sphingomyelin 24:0, ceramides 22:0 and 24:0, and triglycerides; and increases LysoPC 16:0, PC, TMAO, total ceramides, and SM 16:0, 18:0, and 18:1, and ceramide 24:1. These metabolite changes correlated with cardiometabolic outcomes.	–	NCT04127240	(155)
Cross-sectional survey	n=1981 men; angina pectoris;	169-item food-frequency questionnaire and food-composition tables. Self-reported choline intake.	Increased intake of energy-adjusted choline, PC, and sphingomyelin is associated with a higher risk of incident acute myocardial infarction.	85.2	–	(135)
Dietary supplementation. Double-blind, randomized. placebo-controlled	n=97; healthy; >60 y.o.	Groups: 1) 120mg/day Ginkgo Synergy + 700mg/day Choline (n=33; 3 tablets b.i.d.); 2) 100 mg/day OPC Synergy + Catalyn (n=31; 3 tablets b.i.d.); 3) Placebo (n=33; cellulose pills). Cognitive and immune functioning assessment (for 6 months).	No change in IL-2, IL-6, IL-8, IL-10, IL-1α, IFN-γ, TNF-α, VEGF, or MCP-1. 57% decrease in EGF in Group 1 vs placebo.	–	NCT01672359	(173)
Dietary supplementation. Double-blind, randomized, controlled cross-over study.	n=37; healthy men; n=4 non-pregnant women; 21-50 y.o.	Standardized meal containing 600 mg choline as 1) choline bitartrate, 2) PC, 3) no choline. Measure TMAO and choline concentrations in blood and urine at 30 min, and 1, 2, 4, and 6 h; and the gut microbiota composition.	Choline bitartrate yielded 3 times greater plasma TMAO AUC and 2.5 times greater urinary TMAO change from baseline compared to control and phosphatidylcholine groups. Gut microbiota composition differed between high-TMAO producers and low-TMAO producers. High-TMAO producers had more abundant lineages of *Clostridium* from *Ruminococcaceae* and *Lachnospiraceae* compared to low-TMAO producers.	7	NCT04255368	(146)
Dietary supplementation. Randomized study.	n=76; asthma; 15-45 y.o.	Groups: 1) choline supplementation (1500 mg/b.i.d.) + standard pharmacotherapy (inhaled steroids and long-acting β-agonist); 2) standard pharmacotherapy; 6 months.	Choline supplementation decreased symptom/drug score, IL-4, IL-5, TNF-α, cysteinyl leukotriene and leukotriene B4, and 8-isoprostanes; and reduces bronchial hyperresponsiveness, compared to the standard pharmacotherapy group.	–	–	(100)
Dietary supplementation. Cross-sectional study.	n=664; NAFLD. Children (n=114); 9-13 y.o.; Men (n=240); >14y.o.; Premenopausal women (n=116); >19y.o.; Postmenopausal women (n=194).	Demographic categories and intake of choline were recorded during 6 months with baseline and endpoint liver biopsy. Deficient intake is defined as <50% of adequate intake for choline.	Postmenopausal women with deficient choline intake had worse fibrosis (p=0.002). Choline intake was not identified as a contributor to disease severity in children, men, and premenopausal women.	–	NCT00063622 and NCT00063635	(58)
Dietary supplementation. Randomized, double-blind, placebo-controlled trial.	n=84; children, FASDs ; 2.5-5.9 y.o.	Groups: 1) 500 mg choline bitartrate; 2) placebo; 3) 19 mg choline bitartrate /kg. Daily for 9 months.	The choline group has a 12-14% increase in elicited imitation memory paradigm, and 25% increase in memory development. Better response in younger (<4.2 y) than in older (>4.2 y) participants. The 500 mg/day group showed sustained cognitive benefit 4 and 7 years after the intervention.	48-84	NCT01149538 and NCT02735473	(168, 169)
Dietary intervention. Double-blind, randomized, placebo-controlled.	n=36; Type 2 diabetes and mild cognitive impairment; 71.8±5.3 y.o.	Groups: 1) 1200 mg choline alfoscerate/day; 2) placebo; 6 and 12 months.	The choline alfoscerate group increased the mean difference of the Mini-mental State Examination score by +1.7 points, and improved physical health compared to placebo.	–	–	(174)
Dietary intervention. Randomized, Double-blind, placebo-controlled, crossover study.	n=20; resistance-trained young males; 31.3±11 y.o.	Groups: 1) 630 mg alpha-glycerophosphocholine; 2) 315 mg alpha-glycerophosphocholine; 3) placebo.	Both groups taking alpha-glycerophosphocholine improved cognitive performance by the Stroop total score and time of completion.	–	NCT06690619	(175)
Dietary supplementation. Randomized, double-blind, placebo-controlled.	n=53; school-aged children. Prenatal alcohol exposure; 5-10 y.o.	Groups: 1) 625 mg choline/day (n=29); 2) equivalent dose of an inactive placebo (26); 6 weeks.	The Choline group did not improve in cognitive performance in any domain compared to placebo.	6	NCT01911299	(167)
Dietary supplementation. Randomized controlled.	n=40; children and adolescent; NASH; 4-16 y.o.	Groups: 1) Lifestyle modification plus a mix containing docosahexaenoic acid-choline-vitamin E (DHA-CHO-VE, 250mg of DHA, 39 UI of VE, 201mg of CHO); 2) Lifestyle modification plus placebo; daily six months.	DHA-CHO-VE supplementation improved severe hepatic steatosis *(ranging 5-*50%, *p*=0.001*);* ALT and fasting glucose levels. DHA-CHO-VE supplementation did not influence bile acid levels, while increased intestinal FGF19 compared to placebo.	12	NCT01934777	(160)
Dietary supplementation. Randomized cross-over intervention.	n=23; men and women; metabolic syndrome; 35-70 y.o.	Groups: 1) 3 eggs/day; 2) 400 mg choline bitartrate/day; 4 weeks. After a 3-week washout period, allocated to alternate treatment.	During egg phase compared to baseline: increased monounsaturated fatty acids, vitamin D, selenium, and decreased CRP, IL-6 and insulin. During choline bitartrate compared to baseline: decreased IL-6 and not significant trend to decreased insulin.	–	NCT03877003	(162)

**Table 2:** Human studies evaluating choline and choline-related metabodies.

Study	Participants	Intervention	Outcome	Follow-up (months)	Clinical trial #	Ref.
Circulating choline metabolites and the incidence of diabetes	n=3133; free of diabetes at baseline; 33-45 y.o.	Participants from year 15 of follow-up in the Coronary Artery Risk Development in Young Adults (CARDIA) Study. Plasma choline metabolites (choline, betaine, TMAO).	Plasma betaine levels are inversely associated with the 15-year risk of incident diabetes. Plasma TMAO levels are positively associated with the 15-year risk of incident diabetes. Choline was not significantly associated.	–	–	(147)
Circulating choline levels in pulmonary hypertension.	n=272; pulmonary hypertension;	Patients with pulmonary arterial pressure >125 mmHg by right heart catheterization. Fasting plasma samples were assessed for choline levels and clinical variables.	High circulating choline levels (above 12.6 μM) are associated with poor WHO functional class and prognosis, high NT-proBNP, and decreased cardiac output index.	12	–	(140)
Plasma circulating choline as a diagnostic biomarker for hypertension and artery stenosis	n=193; artery stenosis and hypertension; adults.	Plasma choline levels.	High plasma choline in individuals with hypertension and without artery stenosis, and even higher in participants with both hypertension and artery stenosis, compared to healthy controls.	–	–	(154)
Intestinal microbial metabolism of Phosphatidylcholine and Cardiovascular Risk.	n=4007; elective coronary angiography; ~63 y.o. n=40; healthy adults with no chronic illness	Elective diagnostic cardiac catheterization. Measure plasma and urinary levels of TMAO, choline, and betaine after the phosphatidylcholine challenge in healthy participants before and after intestinal microbiota suppression.	Intestinal microbes contribute to increased circulating and urinary TMAO. Major adverse cardiovascular events associated with higher baseline levels of TMAO. High TMAO is a predictor of risk of major adverse cardiovascular events.	36	NCT04255368	(146)
Plasma TMAO association to choline, phospholipids, and methyl metabolism	n=283; healthy and type II diabetes; 66.7±9 y.o.	TMAO and choline plasma concentrations allocated the patients into 4 groups. Group 1: Low TMAO and choline. Group 2: Low TMAO and high choline. Group 3: High TMAO and low free choline. Group 4: High TMAO and choline.	High TMAO and choline (Group 4) are associated with lower mean plasma HDL-cholesterol levels (Group 4=1.33 mmol/L vs Group 1=1.73 mmol/L).	–	NCT02586181 and NCT02588898	(158)
Metabolomics of serum from Systemic lupus erythematosus (SLE).	n=5 men and n=15 women with SLE; n=9 healthy controls. 18-40 y.o. Independent cohort of 38 SLE patients.	Metabolic profiling of human serum.	Serum from SLE patients showed dampened glycolysis, Krebs cycle, fatty acid oxidation, and amino acid metabolism, methyl donors including choline, phosphocholines, methionine, and cysteine. Best discriminators of SLE included elevated lipid peroxidation, gamma-glutamyl peptides, leukotriene B4 and 5-HETE.	–	–	(192)
Lipidomic and metabolomic analysis of serum from SLE.	n=17 SLE and n=17 healthy controls.	Untargeted lipidomics and metabolomics of human serum.	Differential expression of over 50 metabolites, including the elevation of ceramide, TMAO and xanthine in SLE serum, while acylcarnitine, caffeine, hydrocortisone, itaconic acid, and serotonin were downregulated.	–	–	(193)
Serum choline levels in patients with advanced cancers.	INSPIRE cohort: n=106. Advanced solid tumors. LIBERATE cohort: n=51. Solid tumors. Independent validation cohort	INSPIRE cohort: 200mg pembrolizumab, i.v. every 3 weeks (1 cycle). Serum choline levels are measured at baseline and every subsequent cycle. LIBERATE cohort: Anti-PD-1 antibody alone or together with other treatments. Circulating choline levels measured prior treatment and on week 3 and 4.	INSPIRE cohort: No significant changes in absolute neutrophil and lymphocyte count, and neutrophil to lymphocyte ratio (ΔNLR) despite changes in choline levels. No significant differences in T cell numbers (CD8 and CD3) between groups with increased and decreased serum choline levels. Increased choline serum levels, higher levels of CD8^-^ T cells and B cells in the stroma compared to the tumor. CD3^+^CD8^+^ T cells to CD3^+^CD8^-^FOXP3^+^ T cells ratio is significantly higher in the baseline tumors of patients with higher serum choline levels. LIBERATE cohort: ΔANC, Δlymphocytes, and ΔNLR were not significant despite changes in choline levels.	INSPIRE: 12.6. LIBERATE: 11.7.	NCT03702309 and NCT02644369	(235)
Choline serum levels association with cancer risk. Community-based nested case-control study.	n=199; cancer patients. n=199 healthy matched controls. 62.12±6.74 y.o.	Groups according choline quartiles: 1) Q1, lowest quartile (<5.35 µm/mL); 2) Q2: 5.35-14.70 µm/mL; 3) Q3: 14.71–25.18 µm/mL; 4) Q4: highest quartile (≥ 25.19 µm/mL).	Highest choline quartile (Q4) had 3.69-fold increased risk of cancer in the adjusted models (OR = 3.69, 95% CI 1.17-11.63) compared to patients in the lowest quartile. Positive dose-response association between serum choline levels and the risk of overall and digestive system cancer.	3.9	NCT00794885	(204)
Choline-related metabolites in male cancer patients and benign hyperplasia. Case-control study.	n=80; prostate cancer (Pca); 71 y.o. n=51; benign prostate hyperplasia (BPH); 74 y.o.	Measure plasma/serum betaine, free choline, dimethylglycine (DMG), folate, total homocysteine (tHcy), cystathionine, methylmalonic acid, S-adenosyl homocysteine (SAH), S-adenosyl methionine (SAM), and phospholipids before prostate surgery.	No significant differences in choline, betaine, DMG, folate, tHcy, cystathionine, SAH or SAM between the groups. Sphingomyelin species were significantly lower in patients with PCa as compared to the BPH (differences ranged between 6 and 16%).	8	–	(236)

In addition to facilitating nutrient availability in the circulation, diet is a modifiable factor that shapes the composition of the gut microbiome. Different choline sources can exert distinct actions on healthy individuals. In healthy adults (ages 21 to 50), fasted for 10 hours before consuming a standardized meal containing choline bitartrate (free choline), phosphatidylcholine (PC), or a no-choline diet control (NCT04255368) (146), only free choline intake resulted in a three-fold increase in plasma trimethylamine-N-oxidase (TMAO) and a 2.5-fold increase in urinary TMAO compared to control and PC groups. Notably, high-TMAO producers in urine had distinct gut microbiota beta-diversity compared to the low-TMAO producers, characterized by increased lineages of Clostridium species belonging to the Ruminococcaceae and Lachnospiraceae families within the phylum Firmicutes (146). One important unanswered question is how choline-mediated changes in gut microbiome affect intestinal resident immune cells and their capacity to maintain homeostasis and prevent excessive immune responses to commensal bacteria. The organ dysfunction associated with choline deficiency has limited the scope of human studies. As a result, most human research on disease progression has focused on dietary supplementation with different choline sources. Next, we will highlight studies that report associations between choline and inflammatory or immune responses, related molecules, or disease outcomes.

### 3.1 Cardiometabolic diseases

Cardiometabolic diseases include conditions such as diabetes, fatty liver disease, and cardiovascular diseases. Epidemiological studies on circulating choline metabolites and the incidence of diabetes in 3,133 individuals aged 33–45 years found that TMAO levels positively and betaine inversely correlated with the 15-year risk of incident diabetes (147). In a randomized, doubled blind placebo placebo-controlled parallel trial in 96 diabetic patients subjected to dietary supplement intervention that included choline, magnesium or both for 2 months (IRCT20110123005670N25), circulating levels of the inflammation and endothelial factors IL-6 and VCAM-1 decreased significantly in the choline and magnesium group compared to the other groups even after adjusting for potential cofounders (148).

The impact of choline and trimethylamine-N-oxidase (TMAO) on cardiovascular diseases has been investigated in both experimental and human studies. TMAO enhances cholesterol accumulation in macrophages, has been associated with increased cardiovascular disease risk (6, 149), and has a negative impact on disease outcome (150–153). Patients diagnosed with pulmonary hypertension (n=272) with high circulating choline levels (based on the 50th quartile of circulating choline levels, defined as 12.6 µM), exhibit worse key indicators of severe disease progression, including WHO functional class, higher N-terminal pro-B-type natriuretic peptide levels, and reduced cardiac output index, and predicts their prognosis (140). Plasma choline as a diagnostic biomarker for hypertension and artery stenosis was evaluated in 193 individuals, revealing that plasma choline levels were high in patients with hypertension without artery stenosis, and even higher in patients with hypertension with artery stenosis compared to healthy controls (154). The study of the impact of the Dietary Approaches to Stop Hypertension (DASH) diet on plasma choline, choline metabolites, and ceramides in obese older adults revealed a connection between choline metabolites and cardiometabolic outcomes (NCT04127240) (155). The participants in this study consumed either 3 or 6 oz of beef, with rich choline content, within a standardized DASH diet for 12 weeks (155). In response to the DASH diet, with beef intake groups combined, significant changes were observed in plasma biomarkers, including the decreased of plasma choline (by 9.6%); dimethylglycine (10%); phosphatidylcholine (PC) (51%); and triglycerides (18%); and the increase of total lysoPC (by 281%); TMAO (26.5%); total ceramide (22.1%). Around 20 LysoPC species were significantly increased, with lysoPC 16:0 being the most pronounced response. In addition, sphingomyelin 16:0, 18:0, and 18:1 increased by 10.4%, 22.5%, and 24%, respectively, and ceramide 24:1 by 36.8%; whereas sphingomyelin 24:0 significantly decreased by 10%, and ceramides 22:0 and 24:0 declined by 27.6% and 10.9%, respectively (155). These changes in choline and choline metabolites correlated with cardiometabolic outcomes, underscoring the importance of choline in older humans and the role of diet in modulating circulating lysoPC, sphingomyelin, and ceramide species (155). Recently, the study of the effects of L-alpha glycerophosphocholine, a nutritional supplement that has been demonstrated to improve neurological functions, on cardiovascular events in mice has revealed that glycerophosphocholine increases the risk of stroke by shifting the gut microbiota towards abundant Parabacteroides, Ruminococcus, and Bacteroides, while reducing the abundance of Akkermansia, Lactobacillus, and Roseburia (156). These changes reflected an increased relative abundance of choline TMA lyase (cutC). Moreover, glycerophosphocholine supplement also increased the proinflammatory effectors CXC13 and TIMP-1, and activated NF-κB and MAPK signaling pathways in human coronary artery endothelial cells (156). In mice, oral glycerophosphocholine supplementation promotes increased plasma TMAO, phenylalanine, betaine, leucine, and valine (156).

The incidence of acute myocardial infarction was examined in 1981 male patients with stable angina pectoris follow-up during 7.5 years, and subjected to a 169-item food frequency questionnaire to monitor the dietary choline intake, showed that increased intakes of energy-adjusted choline, PC, and sphingomyelin were associated with a higher risk of incident acute myocardial infarction (135). Similarly, a study of 4007 participants (average age of 63) undergoing elective diagnostic cardiac catheterization (NCT04255368) revealed higher baseline TMAO levels in individuals experiencing major adverse cardiovascular events (5.0 μM) compared to those individuals without events (3.5 μM) (157). Participants in the highest TMAO quartile (>6.18 μM) had a 2.54-fold increased hazard ratio compared to controls (157). The prognostic value of elevated plasma TMAO for cardiovascular risk remained significant in various subgroups associated with a reduced overall risk of major cardiovascular events. Notably, a three-year follow-up confirmed elevated plasma TMAO as a significant predictor of major adverse cardiovascular events (157). Another study on cardiometabolic risk factors in a diabetes case-control study (NCT02588898) and a vitamin-supplementation trial (NCT02586181) found high plasma concentrations of metabolites, including TMAO and choline, correlated with lower cholesterol and plasma phospholipid levels, suggesting TMAO may aid in cholesterol solubilization and macrophage cholesterol (158).

Several studies have reported an inverse association between dietary choline supplementation and both the incidence and severity of non-alcoholic fatty liver disease (NAFLD) or steatohepatitis (NASH) (159, 160). Steatosis, or fat accumulation in the liver, which is a direct effect of choline deficiency, can lead to inflammation and cause more severe conditions like fibrosis, cirrhosis and liver cancer (161). Findings from the Framingham Heart Study, a large community-based cohort including offspring and third-generation participants, revealed that choline intake, calculated as the sum of dietary choline-containing compounds including phosphocholine, sphingomyelin, free choline, glycerophosphocholine, and PC, was inversely associated with NAFLD risk (159). A randomized controlled clinical trial (NCT01934777) involving children with non-alcoholic steatohepatitis (NASH) evaluated the effects of combined supplementation with docasahexaenoic acid, choline, and vitamin E (DHA-CHO-VE) (160). Participants underwent a lifestyle counseling along with either a daily supplement containing DHA-CHO-VE (250 mg of DHA, 39 UI of VE, 201 mg of choline) or placebo for six months. The DHA-CHO-VE group showed a marked reduction in severe hepatic steatosis (ranging from 5-50%), alongside improved serum ALT and fasting glucose levels (159). While DHA-CHO-VE supplementation did not affect bile acid concentrations, it did increase intestinal fibroblast growth factor 19 (FGF19), a key regulator of bile acid synthesis and metabolism (160). The role of dietary choline supplementation in adult patients with metabolic syndrome was examined in a randomized crossover intervention in 23 individuals supplemented with either 3 eggs/day or 400 mg choline per day for 3 weeks, followed by a 4-week washout, and then the alternate interventions (NCT03877003) (162). During the egg-derived choline feeding phase, there was a stronger effect on metabolic and inflammatory plasma biomarkers, including an increase in monounsaturated fatty acids, vitamin D, selenium, and a decrease in CRP, IL-6, and insulin (162), while during choline bitartrate feeding, only a decrease in IL-6 was observed (162). This distinct effect may be caused by differences in the lower intestinal absorption of choline bitartrate compared to the choline from eggs (including egg-PC), or by the effect of additional nutrients in the eggs. Collectively, these findings support the idea that different sources of choline have distinct effects, suggesting that a more personalized selection of choline forms, based on individual’s underlying conditions and characteristics, may lead to greater health benefits.

### 3.2 Cognitive decline and Alzheimer’s disease

Recent human and animal research has emphasized the importance of choline intake in preventing neurodegenerative diseases such as Alzheimer’s disease (163). High choline intake in early life has been shown to improve outcomes in a mouse model of Alzheimer’s disease, regulating hyperexcitability, preserving hilar neurons, and enhancing spatial memory (164). Further evidence comes from studies using the 3xTg-Alzheimer’s disease mouse model, which replicates key features of human Alzheimer’s disease progression. In this model, choline-deficient diet from 3 to 12 months of age disrupted liver and heart normal function, and altered neural networks associated with microtubule stability and postsynaptic membrane regulation in the hippocampus, elevated soluble and insoluble Amyloid-β levels, increased Thioflavin S structures, and tau hyperphosphorylation at various pathological epitopes in the hippocampus and cortex (163). This system-wide dysfunction in mice fed with choline-deficient choline was observed in the circulation, as it modulates plasma proteins associated with inflammation, immune response, and metabolic processes, including insulin metabolism, mitochondrial function, inflammation, and fructose metabolic processing (163). Lifelong choline supplementation in another Alzheimer’s disease mouse model (APP/PS1 transgenic mice), significantly diminished amyloid-β plaque load and decreased activated microglia, thereby mitigating the detrimental effects of brain inflammation associated with Alzheimer’s disease (44). The use of the cholinergic neurotransmission-enhancing agent choline alphoscerate has also shown protection from antibody-mediated neurotoxicity, directly activating the a7nAChR receptor in microglia, leading to phenotype switching towards a less inflammatory state (165). Human studies further support the neuroprotective role of choline. A study with 3,224 participants found that low choline intake was associated with an increased risk of incident dementia and Alzheimer’s disease (136). Similarly, a study with 125,594 participants showed that moderate dietary choline intake, ranging from 332.89 mg/d to 353.93 mg/d, is associated with lower odds of dementia and better cognitive performance (166).

In the context of fetal alcohol spectrum disorders (FASDs), 625 mg of choline was administered to school-aged children (5–10 years old, n=29 choline and n=26 placebo) for six months (NCT01911299), showing no effect on cognitive performance (167). However, in another study in younger children with weight-adjusted dosing, the dietary choline intake increased the memory scores by 12-14% (NCT01149538 and NCT02735473) (168, 169). Indeed, an inverse relation between choline dose (mg/kg) and memory improvement suggested weight-adjusted doses as preferable to fixed doses (168). Follow-up studies for 4 and 7 years after the intervention with 500 mg/day for 9 months showed that sustained cognitive benefit was associated with potential improvements in associated white matter microstructure in the choline group (170, 171), which suggests that choline supplementation has the potential to alter brain architecture. The participant in the group supplemented with choline showed an 12-25% increase of the elicited imitation memory paradigm, 8% higher verbal IQ, 29% higher visual-spatial reasoning, 36% higher crossmodal learning, 27% higher non-verbal working memory 4 years after the treatment at a mean of 8.6 year of age. Moreover, an improvement in lower-order executive function skills (eg, information processing speed) was found in a subset of participants who returned 7 years after completing a choline trial, at a mean age of 11 years (170, 171). While these findings are promising, other studies in pediatric patients (aged 7–12 years) diagnosed with ADHD did not show statistically significant differences between the use of citicoline (cytidine diphosphate-choline supplement) and placebo. If the absence of effect is due to the choline form used, or the age of the individual is not known (172).

In the adult population, a double-blind randomized clinical trial examined choline dietary supplementation on cognitive and immune function in 97 healthy older adults over six months (NCT01672359) (173). The participants were divided into three groups: group 1 (n=33) received 120 mg/day of Ginkgo Synergy plus 700 mg/day of choline; group 2 (n=31) received 100 mg/day OPC Synergy plus Catalyn; group 3 (n=33) received a placebo (173). While no significant changes were observed in cytokine levels (IL-2, IL-6, IL-8, IL-10, IL-1α, IFN-γ, TNF-α, VEGF, and MCP1), the Ginkgo Synergy plus choline group exhibited a significant reduction (57%) in epidermal growth factor (EGF), a protein that is often overexpressed in individuals with mild cognitive impairment or Alzheimer’s disease (173). However, the absence of follow-up studies limited the ability to conclude the effect on the disease progression.

The effect on cognitive function has also been examined in 36 individuals with Type 2 diabetes mellitus and mild cognitive impairment assessed by the Mini-Mental State Examination (MMSE) score. The group provided 1200 mg/day of choline alfoscerate for 12 months, significantly showed better physical health and an increase mean difference in the MMSE score (+1.7 between the two groups), which would support its use as an adjunct therapy for managing early cognitive decline (174). The effect on physical performance has also been observed in a recent randomized, double blind, placebo-controlled crossover approach in 20 resistance-trained young males (31.3 ± 11 years) who consumed either a placebo, 630 mg alpha-glycerophosphocholine, or 315 mg of alpha-glycerophosphocholine (NCT06690619). Both groups taking alpha-glycerophosphocholine increased the cognitive performance (Stroop total score and time of completion) (175).

### 3.3 Inflammatory and autoimmune diseases

In inflammatory disorders, the increased metabolic demands for immune activation and cellular proliferation necessitate an elevated supply of phospholipids, such as phosphatidylcholine (PC). High plasma and tissue choline concentrations are common in diseases characterized by chronic low-grade inflammation, supporting the role of choline in modulating immune cell activation and tissue damage (176–182).

#### 3.3.1 Autoimmune and degenerative diseases

For instance, in rheumatoid diseases, lipidomic studies in synovial fluid have reported altered phospholipid profiles with enrichment of choline-containing lipids, such as PC (183–186), which correlate with enhanced ChoKα expression and activity. This has positioned ChoKα as a major enzyme involved in the anomalous cellular lipid metabolic profile of inflammatory disorders, such as RA (25). In rheumatoid arthritis (RA), levels of PC increase in response to inflammatory mediators such as TNF-α, PDGF, and IL-1β (68). In RA, Fibroblast-like synoviocytes (FLS) contribute to synovial inflammation by producing inflammatory mediators and recruiting and activating immune cells, and ChoKα is highly expressed in both osteoarthritis and RA synovial tissue and cultured FLS (187). Exposure to inflammatory mediators such as TNF-α and PDGF, increased ChoKα expression and activity in FLS, suggesting activation of this pathway in the RA synovial environment. The inhibition of ChoKα suppressed the pathogenic behavior of RA-FLS, limiting cell migration and resistance to apoptosis, which may contribute to cartilage destruction in RA. *In vivo* evidence for the role of choline metabolism in RA comes from studies using the K/BxN serum-transfer mouse model of inflammatory arthritis, where treatment with the choline kinase alpha (ChoKα) inhibitor MN58b (3 mg/kg) prevented disease onset (68). Notably, when administered after disease establishment, MN58b also drastically reduced joint swelling, supporting the idea that ChoKα inhibition could serve as an effective adjuvant to current RA therapies by targeting the pathogenic activity of FLS (68).

Other recent studies have investigated the lipidomic profile of arthritis patients in different phases of the disease to understand the correlation between lipid alteration and the severity of local inflammation (188–190). Untargeted lipidomics analysis of synovial fluid and serum from RA patients across various clinical stages, ranging from preclinical to active and sustained phases, showed that despite normal erythrocyte sedimentation rate and CRP at pre-clinical stages, the lipidomic profile of preclinical RA joint fluid closely resembled that of active RA (191). Specifically, alterations in a set of lysoPC, PC, phosphatidylethanolamine, and sphingomyelin subclasses correlated with RA activity (189). Indeed, a strong association was found between lipidome profile in the arthritic joint fluids of RA patients and the severity of synovitis. The sensitivity of lipid profiles in reflecting RA activity and response to disease-modifying anti/rheumatic drugs (DMARDs) (191). Therefore, the lipidome profiles may be considered as a potential biomarker tool to predict the progression of preclinical to established RA disease and facilitate monitoring of disease activity and treatment outcomes (191).

In systemic lupus erythematosus (SLE), which is characterized by chronic activation of self-reactive lymphocytes and myeloid cells, untargeted lipidomics using LC/MS and GC/MS has revealed altered serum concentrations of choline, ACh, phosphocholine, and specific species of PC, lysoPC, and sphingomyelin (192–194). The ACh derived from fibroblastic reticular cells in the lymph nodes is an essential regulator of autoreactive B cell responses. In particular, ACh enhanced B cell differentiation into IgG-producing plasma cells by increasing lipid influx via CD36 and boosting mitochondrial respiration and fatty acid oxidation, leading to an autoreactive phenotype (195). Indeed, the hypomethylation of CD40L in T cells has also been associated with increased disease activity in SLE patients (196, 197). In female patients with SLE, the increase of 168 mg choline per day was associated with a 10% higher methylation of CD40L promoter (198), Besides these findings, it has not been evaluated whether different forms of dietary choline can modify disease progression or the frequency of flares in SLE patients, neither if the selective inhibition of ACh signaling in B cells can prevent autoreactivity and the severity of organ-specific manifestations.

#### 3.3.2 Asthma and pulmonary disease

Studies focused on clinical outcomes in asthma patients who received dietary choline supplementation have been reported in small cohorts with opposing results. In a double-blind, placebo-controlled, crossover trial (NCT02371993) (199) on asthma in elderly individuals (n=30 participants aged >65 years), intake of 310 mg choline bitartrate twice daily for six weeks, did not show significant effects on peripheral blood eosinophil count or total serum IgE levels compared to placebo (199). However, in another study asthma patients (ages 15-45) receiving oral choline supplementation (1500 mg b.i.d.) with inhaled steroids (Budesonide; 400 μg twice daily) and long-acting β-agonist (LABA; formoterol fumarate; 6 μg twice daily) for six months (94), required less additional therapy and had improved bronchial hyperreactivity with a reduction in eosinophil count and total IgE, IL-4, IL-5, TNF-α, and airway inflammatory lipid mediators such as Cys-LT, LTb4 and 8-isoprostanes with no significant changes in IL-10 and IFN-γ, compared to the group with standard pharmacotherpay alone (94). Similarly, other studies in this same line have found a positive impact of high choline supplementation in decreasing symptom scores, the number of asymptomatic days (200). Although these findings suggest that choline can be used at higher doses as a prophylactic intervention or adjuvant to standard therapy in the management of asthma, the exact mechanism by which choline attenuates airway inflammation has not been completely explained.

### 3.4 Cancer progression and anti-tumor immunity

The role of choline metabolism in cancer progression and growth has been extensively studied and revised elsewhere (201, 202). Enhanced choline uptake and metabolism, and increased serum choline are hallmarks of many cancers and correlate with a higher risk of overall cancer with a poor prognosis (203, 204). Notably, elevated choline kinase alpha (ChoKα) expression and activity are often associated with malignant transformation, invasion, and metastasis in some human cancers, making ChoKα a promising therapeutic target in oncology and choline radiotracers a reasonable tool for monitoring cancer growth and therapy response (205–213). Moreover, ChoKα has been recognized as a prognostic marker in various cancers (208, 214–216). High ChoKα expression correlates with early-stage non-small-cell lung cancer (NSCLC) patients at risk of recurrence, whereas lower expression identifies patients with favorable outcomes, potentially allowing for less aggressive treatment approaches (215). Over recent decades, numerous ChoKα inhibitors have been developed and tested for cancer therapy (217–219). However, the efficacy and predictive value of choline metabolism-related signatures for patients’ prognosis, immune microenvironment, and chemotherapy response remain incompletely understood. Recent evidence using several public datasets from The Cancer Genome Atlas (TCGA), Kyoyo Encyclopedia of Genes and Genomes (KEGG), AmiGO (2) and Reactome Pathways databases has identified two choline metabolism-related genes (choline kinase β, *CHKB*, and phosphatidylethanolamine N-methyltransferase, *PEMT*) as key genes involved in the pathogenesis of human colorectal cancer (220, 221). Patients were stratified into high- and low-risk groups based on the optimal cutoff value of the choline metabolism-related risk score to assess the prognostic accuracy of the choline metabolism-related signature (222). The overall survival of patients in the high-risk group was significantly worse than that of patients in the low-risk group. The examination of sc-RNAseq revealed that *CHKB* expression was mainly in endothelial cells, while *PEMT* was highly expressed in CD4^+^ and CD8^+^ T cells (220). Indeed, there were notable differences in immune microenvironment composition, immune checkpoint gene expression, and chemotherapy response between the two risk groups (220).

Oncogenic MYC drives aberrant choline metabolism by transcriptionally upregulating CTP:phosphocholine cytidylyltransferase-α (*PCYT1A*) (223), a key enzyme in phosphatidylcholine (PC) *de novo* biosynthesis. In patients with diffuse large B-cell lymphoma (DLBCL), elevated *PCYT1A* expression, accompanied by increased *MYC* levels and decreased serum PC, correlates with a higher international prognostic index risk classification, suggesting that co-expression of *MYC* and *PCYT1A* may serve as a biomarker for disease progression (223). The use of histone deacetylase inhibitors (HDACI) modulates lipid metabolism and survival pathways in DLBCL, particularly by gaining dependency on the choline pathway and PI3K signaling activation (224), which results in a decline in the antineoplastic effects of the HDACI (224). In part, the aberrant choline metabolism in cancer is driven by molecular alterations in enzymes such as phospholipases C and D, ethanolamine kinase-α, glycerophosphocholine phosphodiesterases, and choline transporters (211). Tumors exhibit elevated phospholipid levels, characterized by an increase in phosphocholine and total choline-containing metabolites, along with an altered glycerophosphocholine/phosphocholine ratio (208, 210, 225, 226). As the major phospholipid in eukaryotic membranes, PC is essential for cancer cell proliferation, tumor progression, and invasion (209). Increased total choline signal detected by 1H nuclear magnetic resonance spectroscopy (1H-NMR) is currently being considered as a diagnostic marker in multiple cancers (137, 210, 227, 228). Choline plays a critical role in cancer diagnosis and monitoring. The use of (11) C-choline positron emission tomography/computed tomography (PET/TC) has provided insights into choline metabolism in tumors, including lung, liver, ovarian, and prostate cancers (216, 229–234). Both (1)H-NMR and choline PET imaging are being explored to evaluate treatment responses. The increased expression and activity of choline transporters and enzymes, such as CTL1 and ChoKα, respectively, have led to the development of radiolabeled choline analogs as PET imaging tracers (211).

In the INSPIRE cohort of 106 patients with advanced solid tumors treated with pembrolizumab (anti-PD-1 antibody) (NCT03702309) (235), higher serum choline levels were associated with augmented CD8^-^ T cells and B cells in the tumor stroma, as well as an elevated CD3^+^CD8^+^ cytotoxic T cells to CD3^+^CD8^-^FOXP3^+^ T regulatory cells ratio in the tumors (235). A validation study on 51 patients receiving anti-PD-1 therapy alone or in combination (NCT02644369) (235), found no association between choline levels and changes in absolute neutrophil count (ΔANC), lymphocytes, and neutrophil-to-lymphocyte ratio (ΔNLR) (235). Notably, higher serum choline levels correlated with improved progression-free survival and a trend toward better overall survival (235). A case-control study (NCT00794885), suggested that high serum choline levels were linked to overall cancer risk, particularly among older male smokers (204). A case-control study investigating the differences in circulating concentrations of choline metabolites between elderly men with prostate cancer or benign prostatic hyperplasia (236). While no significant differences were found in choline, betaine, dimethylglycine, folate, total homocysteine, cystathionine, methylmalonic acid, S-adenosylhomocysteine (SAH), S-adenosylmethionine (SAM), the presence of 11 sphingomyelin species was significantly reduced in patients with prostate cancer as compared to benign hyperplasia (236).

In triple-negative breast cancer, a very aggressive disease with a poor prognosis, the commensal microbiota Clostridiales and its metabolite trimethylamine-N-oxide (TMAO) colonize the mammary gland, being more abundant in tumors exhibiting an activated immune microenvironment (237). TMAO was identified as a driver of antitumor immunity, providing a foundation for potential TMAO-based therapeutic strategies. Patients with higher plasma TMAO levels achieved better responses to immunotherapy due to TMAO-induced pyroptosis in tumor cells and enhanced CD8^+^ T cell-mediated antitumor immunity (237). An unbiased metabolic screening using liquid chromatography-tandem mass spectrometry identified TMAO, as a key gut microbiome-derived metabolite that enhances antitumor immunity in pancreatic ductal adenocarcinoma (PDAC) (238). Administration of TMAO, either intraperitoneally or via dietary choline supplement, in PDAC-bearing mice significantly reduced tumor growth. This antitumor effect was associated with a shift toward an immunostimulatory tumor-associated macrophage (TAM) phenotype and an enhanced effector T cell response within the tumor microenvironment, mediated by activation of the type I Interferon pathway (238). Consistently, delivering intravenously TMAO-primed macrophages yields similar tumor-suppressive effects (238). Indeed, combining TMAO with immune checkpoint blockade therapy, such as anti-PD1 and anti-TIM3 antibodies, further reduced tumor burden and improved survival compared to monotherapies alone. Additionally, higher levels of gut bacteria expressing CutC, the enzyme responsible for generating trimethylamine, correlated with longer survival in PDAC patients and enhanced responses to anti-PD1 immunotherapy in melanoma patients (238). Collectively, these findings highlight TMAO as a promoter of antitumor immunity across multiple cancer types. By enhancing immune cell function, reshaping the tumor microenvironment, and boosting responses to immunotherapy, TMAO holds promise as a potential adjuvant to improve the efficacy of cancer immunotherapy.

### 3.5 Sepsis

Sepsis is a life-threatening clinical condition arising from an excessive immune response to infection, leading to high morbidity and mortality rates (239). A major challenge in intensive care medicine is managing severe infections with multiple organ dysfunction, commonly referred to as sepsis shock (240). The Systemic Inflammatory Response Syndrome (SIRS) is triggered by endotoxin, which stimulates the release of inflammatory cytokines, such as TNF-α; IL-1α/β, and IL-17 into the circulation. These cytokines, in turn, prompt hepatic cells to release acute-phase proteins such as CRP for immunological regulation (241). Matrix metalloproteinases (MMPs) are up-regulated by pro-inflammatory cytokines, as well as by acute phase proteins like serum amyloid A (SAA), with counter-regulatory inhibition by IL-4 and IL-13, and released into the circulation from damaged vascular endothelium. An increased MMPs/tissue inhibitor MMPs (TIMPs) ratio is more strongly associated with tissue response to LPS-linked injury than with the acute-phase reaction (242, 243). Choline-deficient diet has been linked to increased hepatic injury and mortality in endotoxemic shock (239). In a canine model of sepsis induced by intravenous injection of 0.2 mg/kg LPS from *E. coli*, choline intravenous administration suppressed the elevation of circulating MMPs and TIMPs, preserved serum IgG and IgM levels, and was associated with reduced acute-phase reaction and multi-organ failure (244).

In a study using the gold-standard experimental model of polymicrobial sepsis, the cecal ligation and puncture (245), metabolic analysis of plasma and urine identified 14 plasma and 11 urine metabolites related to central carbon and choline metabolism (including choline, betaine, methylamine, and creatinine). These metabolites significantly differed between septic mice with and without signs of acute kidney injury (AKI), as defined by Kidney Disease Improving Global Outcomes (KDIGO), as creatinine levels tripled the average of sham mice. These were further validated in 7 pediatric patients with severe sepsis and AKI, and 13 with sepsis with stage 1 or no AKI. Plasma analysis revealed 10 differentially expressed metabolites more abundant in sepsis-associated AKI patients, including succinate, pyruvate, lactate, betaine, dimethylglycine, and choline, resembling the findings in mouse plasma (245). These metabolic changes were supported by alterations in the gene expression of choline metabolism-related enzymes, such as choline dehydrogenase (*Chdh*), betaine-homocysteine methyltransferase (*Bhmt*), and DMG dehydrogenase (*Dmgdh*), and glycerophosphocholine phosphodiesterase 1 (*Gpcpd1*). Notably, flavin-containing monooxygenase 3 (*Fmo3*), the enzyme responsible for TMAO production in the liver, was reduced in sepsis-associated AKI. Mice that received choline intraperitoneally before and during sepsis exhibit lower levels of plasma blood urea nitrogen (BUN) and creatinine, as well as urine neutrophil gelatinase-associated lipocalin (NGAL) levels compared to septic mice treated with vehicle. While these findings support that choline may protect the kidneys during sepsis, the survival rate was not affected by choline supplementation (245). A metabolic study using NMR spectroscopy found distinct metabolic profiles between sepsis survivors and non-survivors, those who succumbed at day 0 of hospitalization. Non-survivors exhibited significantly higher levels of creatine, phosphocreatine, choline, betaine, tyrosine, histidine, and phenylalanine compared to survivors (246). However, another study reported that individuals with sepsis had lower concentrations of phosphatidylcholine (PC), phosphatidylserine, lysophosphatidylethanolamine, and lysoPC but higher creatinine and C17-sphinganine, compared to healthy controls (247).

Animal models have demonstrated that choline supplementation improves survival and reduces TNF-α production (248). In endotoxin-infused mice, free serum choline levels dropped by 49% at low endotoxin administration but increased by 98% within 48 hours after high endotoxin exposure (1 mg/kg). Despite these fluctuations, phospholipid-bound choline levels increased regardless of the endotoxin dose, accompanied by an increase in biochemical markers of tissue injury and organ dysfunction (249). However, another study using the CLP mouse model found that administering choline before and after sepsis initiation reduced serum inflammatory mediators such as TNF-α and HMGB1, and improved survival via the α7nAChR-dependent mechanism (250, 251). The anti-inflammatory effect of choline was confirmed ex vivo, where endotoxin-activated human whole blood and macrophages exposed to supraphysiological choline concentrations (between 1–50 mM) exhibited reduced TNF-α production (250). Additionally, elevated plasma choline acetyltransferase (ChAT) concentrations were observed in patients with sepsis, with the highest levels detected in those who succumbed to infection (252). The increase in circulating ChAT coincides with the decline in TNF-α (252), showing its potential anti-inflammatory. However, ChAT activity did not correlate with circulating choline or acetylcholine (ACh) concentration, which suggests additional regulatory mechanisms.

Sepsis is characterized by profound metabolic alterations, with choline metabolism playing a key role in inflammatory regulation and organ function. While experimental and clinical data suggest that choline supplementation may protect against sepsis-associated AKI and modulate inflammation, its effect on overall sepsis survival remains inconclusive. Further research is needed to determine the therapeutic potential of choline in sepsis management, particularly in modulating immune responses and metabolic homeostasis.

## 4 Conclusion

Choline metabolism plays a critical role in shaping both the innate and adaptive immune responses, with its effect varying by cell type, metabolite state, and immune environment. While choline shows therapeutic potential, such as reducing inflammation and supporting cognitive and liver health, it can also contribute to disease, including increased risk of cardiovascular events or cancer progression. Despite the growing interest in examining the specific impact of choline on the activation and function of immune cells, the precise downstream molecular signaling and pathways regulated by choline availability remain elusive. Collectively, animal and human studies investigating dietary choline supplementation emphasize the idea of divergent effects based on the choline source, the subject’s genetic and physiological characteristics, and the tissue or cell type studied, suggesting that a more context-specific selection of choline forms may lead to greater health benefits.

Future clinical studies should account for baseline choline status, microbiome profiles, genetic polymorphism in choline metabolic pathways, and organ-specific outcomes to define optimal strategies for leveraging the use of modulating choline availability and metabolism in personalized therapeutic interventions.
